# High Resolution Methylome Map of Rat Indicates Role of Intragenic DNA Methylation in Identification of Coding Region

**DOI:** 10.1371/journal.pone.0031621

**Published:** 2012-02-15

**Authors:** Satish Sati, Vinay Singh Tanwar, K. Anand Kumar, Ashok Patowary, Vaibhav Jain, Sourav Ghosh, Shadab Ahmad, Meghna Singh, S. Umakar Reddy, Giriraj Ratan Chandak, Manchala Raghunath, Sridhar Sivasubbu, Kausik Chakraborty, Vinod Scaria, Shantanu Sengupta

**Affiliations:** 1 CSIR-Institute of Genomics and Integrative Biology, Delhi, India; 2 National Institute of Nutrition, Hyderabad, India; 3 CSIR-Centre for Cellular and Molecular Biology, Hyderabad, India; UCLA-DOE Institute for Genomics and Proteomics, United States of America

## Abstract

DNA methylation is crucial for gene regulation and maintenance of genomic stability. Rat has been a key model system in understanding mammalian systemic physiology, however detailed rat methylome remains uncharacterized till date. Here, we present the first high resolution methylome of rat liver generated using Methylated DNA immunoprecipitation and high throughput sequencing (MeDIP-Seq) approach. We observed that within the DNA/RNA repeat elements, simple repeats harbor the highest degree of methylation. Promoter hypomethylation and exon hypermethylation were common features in both RefSeq genes and expressed genes (as evaluated by proteomic approach). We also found that although CpG islands were generally hypomethylated, about 6% of them were methylated and a large proportion (37%) of methylated islands fell within the exons. Notably, we obeserved significant differences in methylation of terminal exons (UTRs); methylation being more pronounced in coding/partially coding exons compared to the non-coding exons. Further, events like alternate exon splicing (cassette exon) and intron retentions were marked by DNA methylation and these regions are retained in the final transcript. Thus, we suggest that DNA methylation could play a crucial role in marking coding regions thereby regulating alternative splicing. Apart from generating the first high resolution methylome map of rat liver tissue, the present study provides several critical insights into methylome organization and extends our understanding of interplay between epigenome, gene expression and genome stability.

## Introduction

The genome *per se* appears to be static, incorporating stable changes in sequence in spans of generations. However, higher organisms require versatile characteristics in order to maintain homeostasis with their fluctuating environmental niche [1], [2]. To cater to such needs, mechanisms of chemical modifications of chromatin have evolved, that retains the genetic code but transiently alter its functional potential [2]. Such modulations are termed epigenetic modifications; key modifications include acetylation/methylation of histones and methylation of cytosine bases [1]. Aberration/dysregulation of these epigenetic signatures influences the transcription and leads to altered protein expression. The regulation and characteristics of DNA methylation remains enigmatic although it has been implicated in a range of processes like genomic integrity, X chromosome inactivation, genomic imprinting, transposon silencing and diseases like cancer, cardiovascular diseases, etc [3]–[7]. Thus, for a comprehensive understanding of these processes and manifestation of related diseases along with their prognosis, it is imperative to investigate the distribution pattern of DNA methylation at genomic level [4]–[6].

With the advent of newer technologies, elucidation of methylation profiles as a function of the genome is now possible. In this regard, immunoprecipitation of methylated DNA by monoclonal antibodies specific to 5-methylcytidine (5mC) (MeDIP) followed by microarray analysis (MeDIP-Chip) or direct sequencing (MeDIP-Seq) has been used as a valuable tool to map methylated DNA on a genomic scale [8], [9]. The MeDIP-Seq approach provides sequence-level information that aids in distinguishing highly similar sequences as opposed to MeDIP-Chip (using microarrays) where technical drawbacks of cross hybridization, prior knowledge for probe design and low sensitivity from poorly methylated regions limits its use in the study of whole genome methylation [10]–[12]. However, unlike whole genome bisulfite sequencing which provides single base resolution of methylated cytosines, MeDIP-Seq gives sequences of the region that are enriched in methylation [13].

A significantly lower cost and ease of data analysis makes MeDIP-Seq an attractive method to study tissue or cell specific genome-wide methylation profiles [13]. Such studies using model systems have revealed some unique features of the methylome landscape like promoter hypomethylation and gene body hypermethylation [14]. In the context of model systems, human along with mice and rat (*Rattus norvegicus*) forms a triumvirate which has been extensively used to study various aspects of mammalian biology [15]. However, although a number of human pathologies have been probed in terms of its epigenetic component using mice and human, reports based on rat are barely handful [6], [16]–[18]. For diseases like cancer, cardiovascular diseases, neurological disorders, etc where rat is the primary animal model to investigate physiological alterations, a high resolution DNA methylation map is necessary to understand their regulation at molecular level [15]. Moving a step closer to addressing some vital questions to understand the basic methylome structure, we have for the first time generated a high resolution methylome of a rat liver.

Analysis of the rat liver methylome revealed low methylation around transcription start sites (TSS) and high methylation at exons, which is in agreement with previously reported observations in other model systems [19], [20]. We observed that although CpG islands in general had low methylation, some of these islands located mainly within exons were methylated. We also observed that intron/exon boundaries had a distinctive methylation pattern with the terminal exons (UTR's) being lowly methylated. However, if even part of these terminal exons contained protein coding region, they were found to be methylated. Similarly, we observed DNA methylation marks on the coding exons and at introns that are classified in intron retention category of alternate splicing. High methylation at introns predicted their inclusion in transcript as validated using Reverse transcriptase PCR (RT-PCR). Our findings support the notion that DNA methylation, either independently or in conjunction with other components of the epigenome, might play an important role in alternate splicing.

## Results

We generated MeDIP libraries enriched for methylated fraction of the rat liver genome by using Illumina's sequencing protocol that was modified according to Down et al [8]. The efficiency of enrichment was checked by real-time quantitative PCR using known imprinted regions and regions lacking CG sites as control for methylated and unmethylated regions respectively. We achieved a significant enrichment efficiency of ∼30–250 folds as shown by an E value of 4.93 to 8.91 (Table S1).

The MeDIP libraries were sequenced on Illumina Genome Analyzer (GAIIx) to generate the first high resolution methylome map of rat liver. We used Mapping and Assembly with Qualities (MAQ) algorithm to assemble the reads onto the reference genome (rn4) downloaded from UCSC Genome Bioinformatics and obtained ∼120 million pass filter reads [21], [22]. From this, we generated 264,454 methylation peak summits using Model-based analysis of ChIP-Seq (MACS) (Excel S1, Figure S1) [23]. Saturation curves showing the depth of sequencing were plotted by a systematic data reduction approach using MACS which confirmed that we had generated sufficient data to cover the whole genome (Table S2; Figure S2). The ∼4.3 GB of sequence data generated in our study was comparable to earlier reports where ∼1.4 GB of sequence data (in humans) was shown to provide sufficient genome coverage in a MeDIP-Seq experiment [24]. Our data showed high degree of concordance between two replicates as evident from Figure S3. The correlation of methylation peaks (r = 0.84) obtained in our study was similar to that reported in other studies [25]. We independently validated the results obtained in MeDIP-Seq by randomly sequencing a few methylated and unmethylated regions after bisulfite conversion and cloning (Figure S4).

### DNA methylation framework of the *Rattus norvegicus* genome

**Figure 1:**
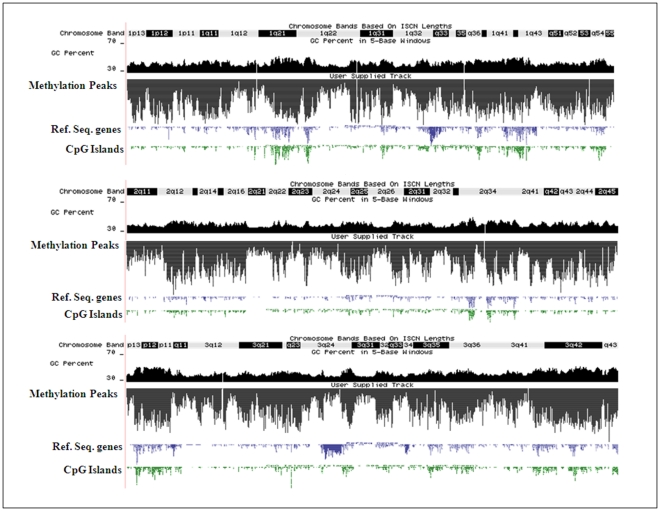
Chromosomal distribution of DNA methylation. Graphical representation of chromosome wide distribution of methylation peaks of chromosome 1, 2 and 3 along with their GC percentage (dark black color), Refseq genes (blue color), CpG Islands (green color), and chromosome band in UCSC Genome Browser.

Individual chromosomal distribution of methylation as a function of GC percentage, RefSeq genes (16,908) and CpG Islands (15,302) in rat genome (data downloaded from UCSC Genome Bioinformatics) is shown in File S1 and representative figures for three chromosomes (1,2 and 3) are shown in Figure 1.

**Figure 2:**
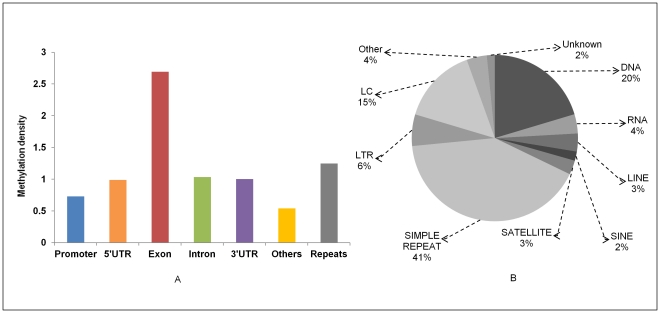
Methylation density in different genomic regions. Methylation density within promoter, gene body and repeats was calculated by dividing the peak summit count in that region by the area of that region. Further repeats were classified in different classes and average methylation level of each class was calculated and plotted.

To ascertain relative methylation in each bin, we performed a detailed analysis by calculating the ratio of methylated peaks located in a particular class (like exons, introns, promoters, repeats, etc) to the total area of that class in the genome (Figure 2A). As repeats occupy a major portion of the mammalian genome we looked at the methylome architecture in context of different repeat classes and found that they account for half (53.3%) of total methylation peak summits encompassing whole genome [26]. We observed differential methylation of repeat elements with high methylation in simple repeats (41%), DNA repeat elements (20%) and low complexity repeats (15%) (Figure 2B). Within the gene body, exons showed higher methylation than introns and UTRs. The average methylation of promoters was found to be the lowest amongst all the classes in the gene body (Figure 2 Figure S5).

### Class distribution of CpG Islands based on methylation

**Figure 3:**
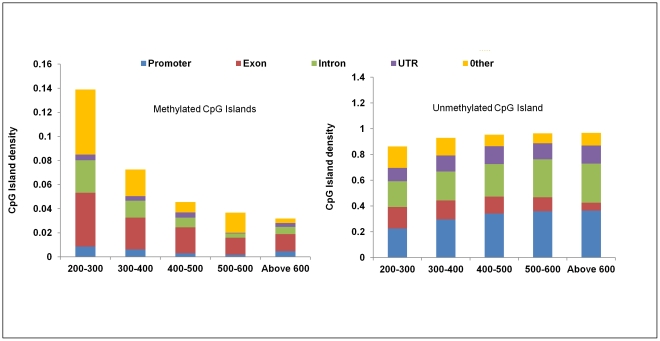
Genomic distribution of methylated and unmethylated CGI. CpG Island of each methylated and unmethylated Islands were classified in different bins on the basis of size. A – Number of methylated CpG Islands in a particular bin was calculated in different regions like intron, exon, promoter (5 kb upstream from the transcription start site) and rest was put in others category. The count was then normalized by the total number of CpG Island in that bin. B – Number of unmethylated CpG Island of bin was calculated in different regions like intron, exon, promoter (5 kb upstream from the transcription start site) and others, and the count was then normalized by the total number of CpG Islands in that bin.

CpG islands (CGI) are pivotal foci for epigenetic modulations, generally believed to be unmethylated, except for the islands located at the genomic imprinting loci and those present on the inactivated X chromosome [3]. However, recent evidences point to the fact that some of the CpG islands may be methylated [27], [28]. We found that of the 15809 CpG islands reported in the UCSC genome bioinformatics for rat, about 6.4% (n = 1020) were methylated (Figure 3). We then categorized both methylated and unmethylated CGIs based on their size (Table S3) and queried the numbers of CGI in each class. We found that a large proportion (48%) of methylated CGI were in the size range of 200–300 bases and the number of CGIs decreased with increase in the size of the islands. Further, we found that methylated CGIs were enriched in exons compared to other classes (37%), while unmethylated ones were mainly present in the promoters followed by exons (Figure 3).

### Promoter and Gene Body Methylation

**Figure 4:**
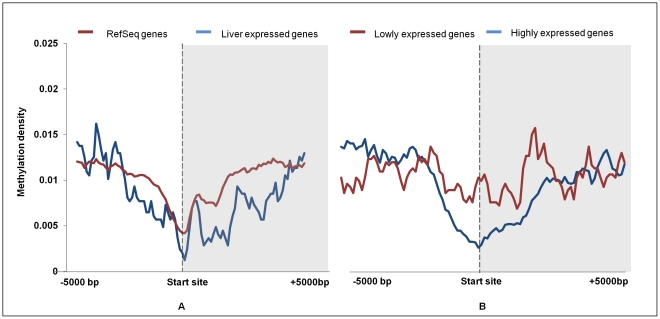
Average methylation density around transcription start site (TSS). A - Distribution of peak summit count in 100 bp sliding window, 5 kb upstream and downstream from the start site was calculated for all RefSeq genes and identified liver proteins. Count was normalized by dividing individual count with total number of genes in that category. The plot obtained of RefSeq and identified liver proteins were further smoothened by taking a moving average of 5. B – Similar distribution of peak summit count in 100 bp sliding window, 5 kb upstream and downstream from the transcription start site was calculated for up regulated and down regulated genes in normal rat liver tissue. Smoothing of peaks was done by taking moving average of 5.

Regulations of genes are known to be affected by methylation in the promoter or in the gene body [29], [30]. As expected, the average methylation pattern at Transcription start site (TSS) in RefSeq genes showed a V shaped curve indicative of low methylation levels at the TSS (Figure 4A). Since all the RefSeq genes are not expressed in a particular tissue, we looked at the methylation pattern around the TSS of highly and lowly expressed genes. To analyze this, we downloaded the data for the genes expressed in liver from a microarray study (GSE19830) [31]. Genes that had expression levels greater or lower than one standard deviation from the mean were considered to be highly or lowly expressed respectively. On plotting the methylation density, we found that the highly expressed genes showed a typical V shape curve at the TSS while lowly expressed ones did not show any such pattern (Figure 4B). To further validate these findings, we employed a high throughput proteomics approach (GeLC-MS followed by mass spectrometry using Orbitrap LTQ) and applying stringent criteria [95% false discovery rate (FDR), with two peptide or one unique peptide hit], we obtained 494 high confidence proteins expressed in the same liver tissue (Excel S2). These expressed proteins also exhibited low level of methylation at their proximal promoter regions (2 kb upstream of TSS) similar to the highly expressed genes. This suggests existence of a differential pattern of promoter methylation (proximal *vis à vis* distal), which may in turn play a crucial role in regulating gene expression.

**Figure 5:**
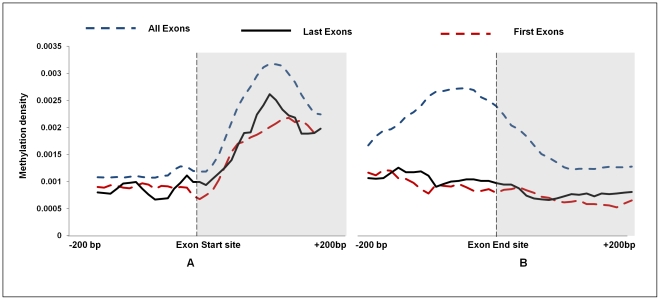
Average methylation density at the intron-exon-intron junctions. Distribution of peak summit count in 10 bp sliding window, 200 bp upstream and downstream from the start site and end site of exons was calculated for all RefSeq gene exons, first exon and all last exons. Smoothing of peaks was done by taking moving average of 5.

Inside the gene body, we observed that exons were more methylated than the introns. At the intron-exon (±200 bases from exon start site)/exon-intron (±200 bases from exon end site) junctions for all RefSeq gene exons, the exon start site was found to be more methylated than the exon end site (intron start site) and there was a sharp transition at the exon boundaries (Figure 5A, B).

To check if the methylation patterns in TSS and exon-intron and intron-exon boundary that we observed in rats are also similar in human and mice, we downloaded the MeDIP-Seq data from the Sequence Read Archive (SRA) and processed it through the same pipeline i.e, peak identification by MACS. We found that the methylation pattern at the TSS of RefSeq genes in human and mice have patterns that are similar to rat methylome data (Figure S6A, B and C). Further, the exon intron junctions also follow a similar pattern in human and mice (Figure S7A, B and C). This is consistent with earlier reports in human embryonic stem cells and neonatal fibroblasts [20]. Thus, DNA methylation may define the splicing boundaries of a gene, thereby helping RNA polymerase II in recognizing exons in a sea of intronic DNA.

**Figure 6:**
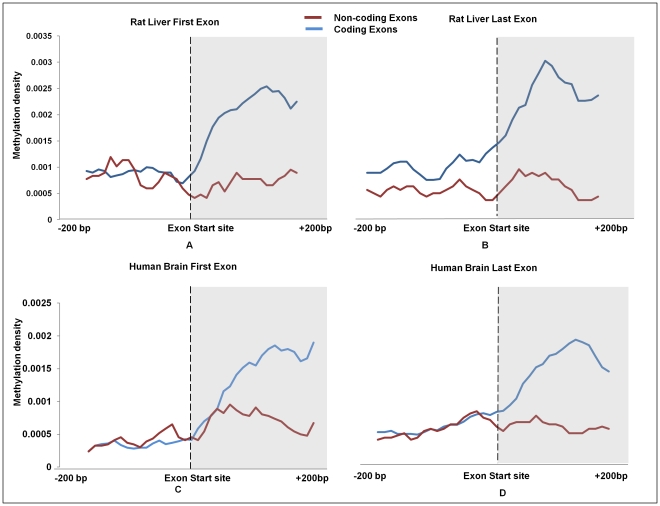
Methylation distribution of first and last exons based on presence and absence of coding region. The first and last exons were further classified as coding exons and non-coding exons based on the fact that they contain coding region within them or not. (a), (b) represents the methylation of rat RefSeq first exon and last exons while (c), (d) represent the methylation pattern in Human RefSeq first exon and last exons plotted using the MeDIP-Seq data from Human brain tissue. Distribution of peak summit count in 10 bp sliding window, 200 bp upstream and downstream from the start site and end site of exons was calculated for first exon and all last exons. Smoothing of peaks was done by taking moving average of 5.

**Figure 7:**
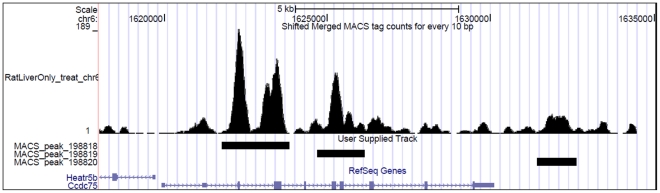
Methylation marks the coding region. Third exon of the *Ccdc 75* gene shows methylation in MeDIP-Seq data as visualized in UCSC genome browser.

**Figure 8:**
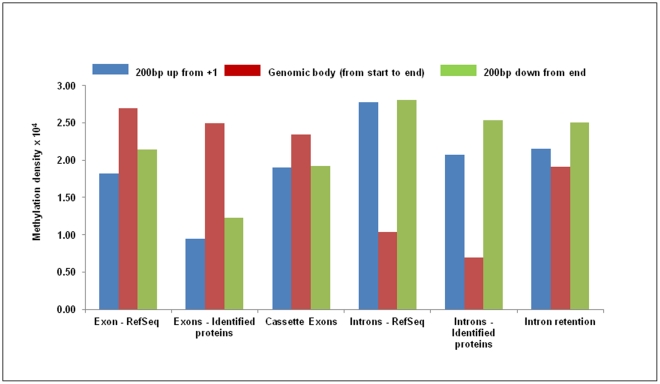
Methylation in alternate splice events. Methylation in genomic features along with Intron retention class of alternative splicing events was calculated. Genomic features include RefSeq exons, introns, identified liver expressed gene exons and introns. Three bins were created: 1) 200 bp upstream from start site of the event, 2) from start site to end of the event, 3) 200 bp downstream from the end. Peak summit count obtained in all bins was normalized by dividing the count with the area of that bin. Distribution of peak summit count in 10 bp sliding window, 200 bp upstream and downstream from the start site of all RefSeq exons and cassette exons.

We observed a marked elevation of methylation at exons which is apparent at the exon start site and similarly the decrease in methylation density at the approach of exon end site. Interestingly, while the first exon also followed the same pattern their methylation density was lower than that generally observed for all RefSeq exons (Figure 5A). The methylation at the beginning of the first and the last exon were lower than other exon (Figure 5A,B). Since, the first and last exons usually constitute the 5′UTR and 3′ UTR regions respectively, we checked methylation levels in first exons and last exons that either contains or lacks coding region and noted that DNA methylation marked the start sites of these exons only if they formed a part of the protein coding sequence (Figure 6A, B). This was further substantiated by the observations that methylation levels up to the second exon were substantially lower in genes where coding starts from the third exon (Figure S8). For instance, in Ccdc 75 gene where coding region starts from the 3^rd^ exon and first two exons, that comprise the UTR, are unmethylated (Figure 7). To confirm that the pattern of cytosine methylation associated with coding part of the transcript is not restricted to rat liver only, we compared methylation levels from human brain tissue, an entirely different tissue type from phylogenetically distant species (Figure 6C, D). Despite significant divergence with respect to tissue and species, we still observe that UTRs are generally not marked by methylation, unless the exon they are a part of, also contains protein coding region. This highlights the conserved nature of DNA methylation pattern in protein coding sequences across species and tissue fates. To prove this point further, we analyzed the methylation status of “introns” that are known to be retained in the transcript (intron retention). We downloaded the data from the Alternative Splicing and Transcript Diversity (ASTD) database, selecting only the unique entries in intron retention (IR) class [32]. We found methylation levels within introns that are retained to be markedly higher compared to constitutive introns that are spliced out (Figure 8; Figure S9A). To confirm that these introns are indeed a part of the mRNA, we performed intron specific RT-PCR in rat liver tissue for a few such introns which were found to be methylated in our data. We isolated RNA from rat liver and converted it into cDNA by RT-PCR using primers that would specifically amplify the introns if they are retained in the transcript (Figure S10A). Similarly, RT-PCR was done for constitutive introns (that were not methylated), which acted as controls. We found that the introns that are methylated are indeed retained in the transcript while the constitutive introns that were unmethylated were not a part of the final transcript (Figure S9B). This strengthens our conclusion that DNA methylation is possibly used as a mark to label the introns as candidates for inclusion within the final transcript by enabling them to evade splicing.

To further prove our point, we analyzed the methylation pattern at the boundaries of cassette exons (of alternative splicing events from ASTD). Interestingly, we observed a marked elevation in methylation density at intron-exon junction in constitutive exons but only mildly higher methylation density at the intronic part of the intron-exon boundary in cassette exons compared to that of the constitutive suggestive of the role of DNA methylation in marking these exons for splicing (Figure 8 Figure S10B). Overall, our observations indicate an important role for DNA methylation in regulation of splicing events and final constitution of the protein sequence.

## Discussion

DNA methylation has two crucial evolutionarily conserved functions. It is one of the subtle control elements that govern gene response to environmental cues and also chief defense mechanism for the genome against selfish mobile elements. Genome-wide methylation map provides a thorough quantitative and qualitative assessment of genomic methylation; a prerequisite for understanding its functional potential both in terms of maintenance of genomic stability as well as gene regulation. There is increasing evidence to show that in addition to its role in X-chromosome inactivation, genomic imprinting, and maintenance of cellular transcriptional memory during development, DNA methylation plays an important role in predicting the course of complex diseases [1], [3], [33].

To understand the importance of DNA methylation in disease causation and progression it becomes imperative to analyze the basal methylome of various tissues in different model systems. Despite rat being a well established model system for studying several complex disorders, methylome from a control rat is not available. Since of complex disorders have a metabolic component and liver is a key regulator of mammalian metabolism, we preferentially choose to look at the liver methylome. To our knowledge this is the first high resolution mammalian liver methylome and it is expected that the rat methylome generated in this study will help the research community to investigate aberrant DNA methylation in several complex disorders for which well established rat models already exist.

Although MeDIP-Seq has several advantages, it runs a risk of generating false positive results especially when raw tags are directly used for assessing methylation levels. We used MACS to sharpen methylation peak summits for better score and thus overcome this problem, MACS improves the spatial resolution of aligned data and imparts robustness to the final alignment of sequences on the basis of a dynamic poisson distribution which corrects for local biases in the genome [23].

Since, this is the first report of rat methylome, we have several interesting observations. We observed that repeat regions in general are highly methylated. Approximately 41.9% of rat genome is covered by repeats which include ten different classes. Repeat elements are usually associated with chromosome instability, translocation and gene disruption via transposition or recombination events [6], [34]. Methylation of repeat elements is known to silence the repeat region and prevent reactivation of endoparasitic sequences [35], [36]. Consequently, repeat regions have been reported to account for a major proportion of genomic methylation which is substantiated by our data. Simple repeats were hypermethylated to a greater extent (∼20% of total methylation peak summits) *vis a vis* other repeat classes and thus might hinder recombination and consequent chromosome instability [37]. LINE (Long Interspersed Nuclear Elements) and LTR (Long Terminal Repeats) were also significantly methylated and accounted for 16% and 11.4% of total methylation peak summits. Methylation of LTRs is crucial in maintenance of genomic stability since transcriptional silencing at these loci via DNA methylation suppresses the initial stages of retrotransposition. A recent study has shown that one endogenous retrovirus (ERV) class i.e., ERV-K-type family contributes to genome variability in inbred rat strains [38]. Therefore, such defence mechanism is important for rodents who harbour active ERV within their genomes.

Although methylation occurs at 70–80% of cytosines that are followed by guanine bases (CpG), there are regions in the genome known as CpG islands (CGI) which are generally believed to be unmethylated in spite of having high GC percentage [3]. These regions are known to play a key role in gene regulation and their aberrant methylation has been reported in various disease conditions such as cancer and several neurological/autoimmune disorders [6]. Our study for the first time provides a catalogue of methylated and unmethylated CGIs in adult rat liver which will aid in better understanding of the disease mechanism in rat models of such diseases. While earlier reports investigating CGI had established hypomethylation as a hall mark of CGIs, a recent report indicates that a small proportion of these islands might be methylated. Straussman et. al. showed that methylated CGIs are generally shorter in size. We for the first time show that these methylated CGIs are significantly enriched in exons and are shorter in size in the rat genome [27]. We also show a distinct methylation pattern upstream of CGIs irrespective of their methylation status with a dip in the methylation levels upstream (∼1 Kb) of CGI start site (Figure S11). Although, this trend is recurrent in rat methylome, we could not identify any underlying specific sequence motif. It is plausible that there are sequence independent messages that bring about this pattern of methylation distribution immediately preceding CGI.

Our data also shows that about 1/3^rd^ of unmethylated CGIs are distributed within the promoter region which are important for expression of genes. Analysis of methylation status across promoter regions of RefSeq genes and expressed genes revealed a V shaped curve, due to declining methylation near TSS which is in agreement with other reports [20], [39]. Expressed genes, like RefSeq genes, were less methylated at proximal promoter region while methylation at distal promoter in both categories was higher. Therefore, collectively the methylation status of proximal promoter diverges from that of the distal promoter. Such a divergence might stem from increased probability of existence of CpG Island at the proximal promoter.

DNA methylation along with nucleosome positioning has been shown to be enriched at the exonic positions in the genome hinting at a role in splicing [40]. During splicing, which generally occurs co-transcriptionally, gene splicing machinery needs to accurately distinguish an exon from an intron [41]. Earlier reports have shown that splicing is influenced by chromatin structure [42]. Our observation that intron-exon-intron junctions are distinctly marked by DNA methylation thus supports the hypothesis that chromatin modification and DNA methylation probably work in tandem to regulate splicing [42], [43]. Choi et al. have shown that the coding margins constituting coding start and end boundaries are demarcated by DNA methylation [44]. However, inclusion of UTR in the final transcript has hitherto remained unexplained. Our analysis revealed that UTR recognition and retention is independent of DNA methylation. In general, UTRs were not marked by methylation unless a part of UTR was included in the coding sequence, in which case they were found to be methylated. A similar pattern was also observed on analysis of human brain tissue, thus, advocating a universal identification code conserved over different species and tissue types. In the light of this observation we suggest that protein coding region of genes harbor distinctly elevated methylation levels in comparison to the non-coding regions, which might help in splicing as postulated by earlier studies [40].

Anastasiadou et al. has recently analyzed a small data set derived from Human Epigenome Project and reported a possible link between methylation and splicing [45]. Our observation of altered methylation in alternately spliced events like cassette exons and intron retention and that of marking of UTR containing coding region with DNA methylation suggests that DNA methylation is possibly used as a mark to label these introns and exons as candidates for inclusion within the final transcript and to enable them to evade splicing. It has been reported that co-transcriptional splicing requires the recruitment of splicing factors at splice sites during transcription, even though completion of intron removal may occur post-transcriptionally [41], [46]. This dynamic link between splicing and transcription has been partially explained by RNA Pol II kinetic model of alternative splicing, which states that recognition of splice sites is dependent on the rate of RNA Pol II elongation [46]. Therefore, it can be perceived that DNA methylation along with chromatin road blocks like nucleosome positioning may cause slowing down of RNA Pol II and lead to alternate splicing. This view is also supported by the fact that nucleosomes and RNA Pol II and DNA methylation (as found in our study) are enriched at the alternate splice sites [46], [47]. Thus, we speculate that DNA methylation may directly or indirectly via nucleosome positioning affect splice site choice and thereby decide the sequence of the final transcript.

Overall, our results show that DNA methylation is one of the marks that a cell employs to distinguish between protein coding and non-coding regions of the genome. Interestingly, methylation seems to mark all the coding exons more than the non-coding ones suggesting presence of an under-appreciated link between DNA modification and coding. While generating the first high resolution methylome map of rat liver, the present study has provided ample intriguing and critical insights into methylome organization and function of cytosine methylation in defining the coding region in a gene. Further studies to validate the functional potential can help identify key methylation signatures in diverse cellular contexts including altered, disease states, which would not only increase our knowledge base but also empower us to design better epigenetic diagnostics.

## Materials and Methods

### Genomic DNA extraction

The experiment was carried out in Wistar rats in accordance with the ‘principles of laboratory animal care’ (US Department of Health, Education and Welfare: Guide for the Care and Use of Laboratory Animals. Washington, DC, U.S. Govt. Printing Office, 1985, (NIH publ. no. 85–23) and with the approval of the ‘Institute's Ethical Committee on Animal Experiments’ at the National Institute of Nutrition, Hyderabad, India. Genomic DNA was isolated from liver of two adult Wistar rats using protocol from Qiagen DNeasy Blood & Tissue Kit (Qiagen, Valencia, USA) and 20 µg/mL RNase was used to degrade the RNA present in the sample. DNA integrity was verified by agarose gel electrophoresis. Quality and quantity of DNA was measured using Nano-Drop Spectrophotometer and Quant-iT PicoGreen dsDNA Reagent and Kits (Invitrogen, USA) respectively.

### Methyl-DNA immunoprecipitation sequencing (MeDIP-Seq)

Before carrying out MeDIP, we sonicated genomic DNA to produce random fragments ranging in size from 100 to 500 bp and purified using the PCR purification kit (Qiagen). Based on the manufacturer's recommended protocol, we then end-repaired, phosphorylated and A-tailed the fragmented DNA and ligated Illumina single read adapters to the fragments. We used ∼4 µg of adaptor-ligated DNA for subsequent MeDIP enrichment. Briefly, following adaptor ligation, DNA was denatured at 95°C for 10 min. Immunoprecipitation was then carried out at 4°C for 3 hr using 10 µg of monoclonal antibody against 5-methylcytidine (Eurogentec) in a final volume of 500 µl IP buffer (10 mM sodium phosphate (pH 7.0), 140 mM NaCl, 0.05% Triton X-100). We incubated the mixture with 40 µl of Dynabeads with M-280 sheep antibody to mouse IgG (Dynal Biotech) for 2 hr at 4°C and washed it seven times with 700 µl of IP buffer. We then treated the beads with proteinase K for 4 hr at 50°C and recovered the methylated DNA by phenol-chloroform extraction followed by ethanol precipitation. PCR amplification by Illumina single read PCR primers was performed as described earlier. We performed agarose gel electrophoresis and excised bands from the gel to produce libraries with insert sizes of ∼200 bp, and quantified these libraries using the Quant-iT PicoGreen dsDNA Reagent and Kits (Invitrogen). We then prepared flowcells with 14 pM DNA using the manufacture's recommended protocol and sequenced for 36 cycles on an Illumina Genome Analyzer II. Obtained images were analyzed and base-called using GA pipeline software version 1.3 with default settings provided by Illumina.

### PCR and real-time PCR on MeDIP samples

We carried out real time PCR reactions with 0.5 ng of input DNA and immunoprecipitated methylated DNA. For real-time PCR reactions, we used the SYBR Green PCR master mix (Kappa Biosystems) and Roche - LightCycler 480 System. For each qRT-PCR reaction (total volume of 10 µl), we used 5 µl SYBR Green PCR master mix and 2 µl primer mix (0.5 µM each). Reaction conditions were as follows: 1 cycle at 95°C for 30 seconds, 35 cycles at 95°C for 30 seconds and 1 cycle at Tm for 30 seconds. Experiments were done in triplicates. We have followed the method described by Tomazou *et al* to evaluate the relative enrichment of target sequences after MeDIP [9]. Briefly we normalized the Ct of the MeDIP fraction to the Ct of the input (ΔCt). Subsequently we normalised the ΔCt of each target sequence to the ΔCt of an unmethylated control sequence (ΔΔCt). Finally, the enrichment was calculated as E = 2^ΔΔCt^. Refer Table S4 for primer sequence

### RT-PCR

Total RNA (1 mg) was isolated using the RNeasy RNA isolation kit (Qiagen) and reverse transcribed using Superscript III (Invitrogen) using random hexamers, according to the manufacturer's protocol. PCR were performed on 1 µl of complementary DNA or a comparable amount of RNA with no reverse transcriptase, using AmpliTaq Gold DNA Polymerase (ABI Biosystems). The list of primers is attached in Table S4.

### Bisulfite Sequencing

DNA (0.5 µg), from same rat liver sample was bisulfite converted using the EZ DNA methylation kit (ZYMO Research) according to the manufacturer's protocol.PCR was performed using primers flanking the methylated and unmethylated regions. PCR products were cloned into pGEM®-T (Promega). Randomly clones were sequenced and then analyzed using BiQ Analyzer [12]. Refer Table S4 for primer sequence.

### Data Download and Analysis

We downloaded the rat genome sequence and mapping information (rn4) from the University of California Santa Cruz Genome Bioinformatics Site (http://genome.ucsc.edu). The reads were mapped onto the rat genome reference sequence using the high-performance alignment software ‘maq’ version 0.7.1 (http://maq.sf.net) and those with maq quality less than 10 were removed from further analysis. We used MACS (version 1.4.0 beta) for peak detection and analysis of immunoprecipitated single-end sequencing data to find genomic regions that are enriched in a pool of specifically precipitated DNA fragments.

The Browser Extensible Data (BED) files of the Human Brain MeDIP seq was downloaded from the SRA012488 [14]. These BED files were then merged and analyzed by MACS to generate peak summit coordinates. The summit files were then used for further downstream analysis. The data for the analysis of alternate splicing events was downloaded from the EBI ASTD database version 1.1 (http://www.ebi.ac.uk/astd/main.htmljsessionid=8E5318CC1D7E9AF0E003465EE3084922).The IPI IDs of identified liver proteins were searched for their gene IDs in ENSEMBLE genome browser and then in UCSC Genome Bioinformatics for gene coordinates. Of the 524 proteins, we could get 494 gene IDs and further analysis was done using these proteins.

For analyzing the methylation pattern between the highly vs lowly expressed genes we downloaded microarray gene expression data for control rat liver from Gene Expression Omnibus (GSE 19830). Data analysis was done using Bioconductor package Affy, via R programming language. Average of the normalized intensities of all three replicates was converted to log base 2, and then statistically highly and lowly expressed (mean ± standard deviation) genes were used to check the methylation pattern across their TSS in a 100 kb sliding window.

The RefSeq genes, repeat element and CGI coordinates of human and rat were downloaded from UCSC Genome Bioinformatics. The CGIs in our study follow the three basic characteristics, a) length greater than 200 bp, b) GC content >50% and c) CpG Observed/Expected >0.6. The methylation status of the CpG Islands was determined by mapping the methylation peak summits (from MeDIP-Seq data) on the CpG islands. Islands having methylation peak summits were designated methylated islands while the rest were termed unmethylated.

For describing the methylation of any event, we have used the term “methylation density”, which in the case of all bar plots is the ratio of methylation peak summit count in the given region to the area in base pairs of that region (Figure 1, 2, 7). While in the case of line plots, methylation density refers to the ratio of methylation peak count vs number of data points (Figure 3, 4, 5).

### 1D SDS-PAGE and In-Gel Tryptic Digestion

Briefly, 100 µg of total protein of rat liver was separated in 12% SDS-polyacrylamide gel using the Biorad SDS-PAGE setup. The protein lane was excised from the gel and chopped into 5 equal fractions. These fractions were digested with trypsin (promega V511A) as mentioned earlier [48].

### Nano-RP-LC MS/MS analysis-

Nanoflow LC MS was performed by coupling a split-free nano LC system (Proxeon nano-LC) with the LTQ Orbitrap mass spectrometer (Thermo Electron, Bremen, Germany). A 3 cm pre-column (100 µm i.d) packed with 5 µm Synergi C18 100Å reverse-phased material was connected via a micro-tee fitted with an electrode for voltage application. This was connected to a 10 cm fused silica microcapillary analytical column (100 µm i.d) with a homemade laser pulled spray tip packed with 5 µm Synergi C18 100 Å reversed phase resin. Each fraction was loaded using a proxeon auto sampler and injected onto a sample storage loop. After equilibrating the columns with 30 ul buffer A at a flow rate of 6 ul/min and 10 ul buffer A at a flow rate of 0.8 ul/min respectively, the sample stored in the loop was loaded onto the trap column for desalting and then onto the analytical column for reverse-phased separation of separation of peptides. A stepwise gradient of the organic phase (Buffer B- 100% and 0.1% formic acid) with a constant flow rate of 300 nl/min was run for a total of 140 min. The composition of the gradient is as follows- 1% Buffer B for 20 min, 45% for 110 min and 100% for 2 min extended to 100% buffer B for 8 min. Nitrogen gas used as sheath (75psi) and auxiliary gas(10 units) gas with the heated capillary at 200°C. CID experiments employed helium with 35% collision energy. The resolution was set to 60000 at positive polarity. The LTQ Orbitrap mass spectrometer was operated in a data dependent MS/MS mode consisting of a full scan at mass range 350–2000 m/z at FTMS mode followed by four data-dependent scans performed in linear ion trap in which the four topmost intense ions was subjected to MS/MS. Dynamic mass exclusion was enabled with a repeat count of once every 30 seconds for a list size of 500.

### Protein Identification and Data Analysis

The .raw spectral files containing MS and MS/MS data were submitted to Proteome Discoverer 6.0 (Thermo Scientific, San Jose, CA) and searched using Sequest algorithm in IPI rat database (IPI.rat.v3.67.). The search was performed against IPI database V3.74 with specified precursor ion mass tolerance of 10 ppm and fragment ion mass tolerance of 0.8 Dalton with 2 missed tryptic cleavages. Oxidation of methionine was set as dynamic modification while carbamidomethylation of cysteine was set as static modification. To eliminate false discovery, the spectra were searched against decoy database 1% targeted and 5% relaxed FDR. The results of all five fractions were combined to give a multi-consensus report.

### Online Data Submission

The MeDIP-Seq data from this study have been submitted to the NCBI Gene Expression Omnibus (http://www.ncbi.nlm.nih.gov/geo/) under accession no. GSE31571. Proteome data and methylation tracks can be accessed from the following links respectively **URL:** http://genome.igib.res.in/epigenome/medip/rat_proteome.rar. and http://genome.igib.res.in/SSG/SSG_MEDIP_rat_liver_control_methylation_tracks.tar.gz.

## Supporting Information

Figure S1 **MACS model for MeDIP-Seq data.** The reads generated from the MeDIP sequencing was processed through MACS (Model-based Analysis for ChIP-Seq) software version 1.4.0 beta for the generation of MACS model. The fragment size was 200 bp and the distance d between the forward and the reverse tags is 38.
(TIF)

Figure S2 **Saturation curve.** The curve shows saturation at the end on plotting the percentage of peak covered by sampling (y-axis) against the percentage of raw reads take during data reduction approach (x-axis).
(TIF)

Figure S3 **Concordance between replicates.** Read coverage at each base pair was calculated separately for both the replicates and then Pearson's correlation was calculated using R programming and statistical language.
(TIF)

Figure S4 **Bisulfite validation of MeDIP Seq data.** Two regions (SSG06 and SSG08) showing high methylation and one region showing no methylation (SSG04) but with a number of CpGs were sequenced after bisulfite conversion and PCR amplification.
(TIF)

Figure S5 **Methylation density in different bins.** Methylation density within genomic features along with all the repeat class (DNA, RNA, LTR, LC, LINE, SINE, SATELLITE, SIMPLE REPEAT, OTHER REPEATS, and UNKNOWN REPEATS) and rRNA (a component of RNA class) was calculated. Genomic features include RefSeq exons, introns, identified liver expressed gene exons and introns.
(TIF)

Figure S6 **CpG methylation distribution around TSS in Human, mice and rats.** Average methylation density around Transcription Start Site (TSS) of 3 different species. Distribution of peak summit count in 100 bp sliding window, 5 kb upstream and downstream from the start site was calculated for all RefSeq genes of A – Human and B - Mouse. Smoothing of peaks was done by taking moving average of 5.
(TIF)

Figure S7 **CpG methylation distribution at exon boundaries in Human, mice and rats.** Methylation density around Exon/Intron junction of 3 different species. Distribution of peak summit count in 10 bp sliding window, 200 bp upstream and downstream from the start site was calculated for all exons of A- Human and B– Mouse. Smoothing of peaks was done by taking moving average of 5.
(TIF)

Figure S8 **Methylation density at the start site of non coding 2^nd^ exons.** Distribution of peak summit count in 10 bp sliding window, 200 bp upstream and downstream from the start site was calculated for all 2^nd^ exons which were non coding. Smoothing of peaks was done by taking moving average of 5.
(TIF)

Figure S9 **Methylation distribution in alternate splice events.** CpG methylation distribution in two different alternate splice events. A: Distribution of peak summit count in 10 bp sliding window, 200 bp upstream and downstream from the start site of all RefSeq exons and cassette exons. Smoothing of peaks was done by taking moving average of 5. B: Distribution of peak summit count in 10 bp sliding window, 200 bp upstream and downstream from the end site of RefSeq exons and introns of Intron Retention class. Smoothing of peaks was done by taking moving average of 5.
(TIF)

Figure S10 **PCR amplification products of alternate splice events.** PCR amplification of introns of IR category showing methylation in our MeDIP-Seq data (A; IRM1 to IRM7) and their constitutive counterparts (B; IRC1 to IRC7).
(TIF)

Figure S11 **Methylated and Unmethylated CGI distribution around TSS.** Methylation pattern in methylated/unmethylated CpG Island around their start site. Distribution of peak summit count in 100 bp sliding window, 5 kb upstream and downstream from the start site was calculated for methylated and unmethylated CpG Islands.
(TIF)

Table S1 **Real Time PCR validation of the MeDIP process.** The methylated regions were selected as imprinted regions either in rat (H19) or in Human and mice (Gnas). Negative regions were randomly taken from the genome where there were no CpGs. The enrichment is shown as E.
(DOCX)

Table S2 **Table showing percentage of reads versus percentage of peaks called by MACS.** Table showing the data generated by MACS employing data reduction approach after the model generation.
(DOCX)

Table S3 **Distribution of CGI based on size.** Distribution of mCGI and uCGI based on their size and genomic location.
(DOCX)

Table S4 **Primer list.** List of primers used in the study: The list contains primers used for calculating MeDIP-Seq enrichment efficiency, for bisulfite PCR and those used for the reverse transcriptase PCR of the rat liver cDNA.
(DOCX)

File S1 **Chromosomal distribution of methylation.** The methylation tracks visualized in UCSC genome browser with CGI tracks and RefSeq genes for all chromosomes.
(DOCX)

Excel S1 **Methylation peak summit file.** Details of each methylation peak generated by MACS.
(XLSX)

Excel S2 **List of proteins found in proteomics study.** Proteins with their accession Ids and related information.
(XLSX)

## References

[1] Bernstein BE, Meissner A, Lander ES (2007). The mammalian epigenome.. Cell.

[2] Szyf M (2007). The dynamic epigenome and its implications in toxicology.. Toxicol Sci.

[3] Bird A (2002). DNA methylation patterns and epigenetic memory.. Genes Dev.

[4] Kelly TK, De Carvalho DD, Jones PA (2010). Epigenetic modifications as therapeutic targets.. Nat Biotechnol.

[5] Petronis A (2010). Epigenetics as a unifying principle in the aetiology of complex traits and diseases.. Nature.

[6] Portela A, Esteller M (2010). Epigenetic modifications and human disease.. Nat Biotechnol.

[7] Sharma P, Kumar J, Garg G, Kumar A, Patowary A (2008). Detection of altered global DNA methylation in coronary artery disease patients.. DNA Cell Biol.

[8] Down TA, Rakyan VK, Turner DJ, Flicek P, Li H (2008). A Bayesian deconvolution strategy for immunoprecipitation-based DNA methylome analysis.. Nat Biotechnol.

[9] Tomazou EM, Rakyan VK, Lefebvre G, Andrews R, Ellis P (2008). Generation of a genomic tiling array of the human major histocompatibility complex (MHC) and its application for DNA methylation analysis.. BMC Med Genomics.

[10] Park PJ (2009). ChIP-seq: advantages and challenges of a maturing technology.. Nat Rev Genet.

[11] Harris RA, Wang T, Coarfa C, Nagarajan RP, Hong C (2010). Comparison of sequencing-based methods to profile DNA methylation and identification of monoallelic epigenetic modifications.. Nat Biotechnol.

[12] Bock C, Reither S, Mikeska T, Paulsen M, Walter J (2005). BiQ Analyzer: visualization and quality control for DNA methylation data from bisulfite sequencing.. Bioinformatics.

[13] Laird PW (2010). Principles and challenges of genomewide DNA methylation analysis.. Nat Rev Genet.

[14] Maunakea AK, Nagarajan RP, Bilenky M, Ballinger TJ, D'Souza C (2010). Conserved role of intragenic DNA methylation in regulating alternative promoters.. Nature.

[15] Jacob HJ (1999). Functional Genomics and Rat Models.. Genome Res.

[16] Acosta D, Suzuki M, Connolly D, Thompson RF, Fazzari MJ (2011). DNA methylation changes in murine breast adenocarcinomas allow the identification of candidate genes for human breast carcinogenesis.. Mamm Genome.

[17] Rosenfeld CS (2010). Animal models to study environmental epigenetics.. Biol Reprod.

[18] Thompson RF, Fazzari MJ, Niu H, Barzilai N, Simmons RA (2010). Experimental intrauterine growth restriction induces alterations in DNA methylation and gene expression in pancreatic islets of rats.. J Biol Chem.

[19] Feng S, Cokus SJ, Zhang X, Chen PY, Bostick M (2010). Conservation and divergence of methylation patterning in plants and animals.. Proc Natl Acad Sci U S A.

[20] Laurent L, Wong E, Li G, Huynh T, Tsirigos A (2010). Dynamic changes in the human methylome during differentiation.. Genome Res.

[21] Li H, Ruan J, Durbin R (2008). Mapping short DNA sequencing reads and calling variants using mapping quality scores.. Genome Res.

[22] Fujita PA, Rhead B, Zweig AS, Hinrichs AS, Karolchik D (2011). The UCSC Genome Browser database: update 2011.. Nucleic Acids Res.

[23] Zhang Y, Liu T, Meyer CA, Eeckhoute J, Johnson DS (2008). Model-based analysis of ChIP-Seq (MACS).. Genome Biol.

[24] Chavez L, Jozefczuk J, Grimm C, Dietrich J, Timmermann B (2010). Computational analysis of genome-wide DNA methylation during the differentiation of human embryonic stem cells along the endodermal lineage.. Genome Res.

[25] Bock C, Tomazou EM, Brinkman AB, Muller F, Simmer F (2010). Quantitative comparison of genome-wide DNA methylation mapping technologies.. Nat Biotechnol.

[26] Mandal PK, Kazazian HH (2008). SnapShot: Vertebrate transposons.. Cell 135: 192–192.

[27] Straussman R, Nejman D, Roberts D, Steinfeld I, Blum B (2009). Developmental programming of CpG island methylation profiles in the human genome.. Nat Struct Mol Biol.

[28] Yamada Y, Watanabe H, Miura F, Soejima H, Uchiyama M (2004). A comprehensive analysis of allelic methylation status of CpG islands on human chromosome 21q.. Genome Res.

[29] Backdahl L, Herberth M, Wilson G, Tate P, Campos LS (2009). Gene body methylation of the dimethylarginine dimethylamino-hydrolase 2 (Ddah2) gene is an epigenetic biomarker for neural stem cell differentiation.. Epigenetics.

[30] Koul S, Houldsworth J, Mansukhani MM, Donadio A, McKiernan JM (2002). Characteristic promoter hypermethylation signatures in male germ cell tumors.. Mol Cancer.

[31] Shen-Orr SS, Tibshirani R, Khatri P, Bodian DL, Staedtler F (2010). Cell type-specific gene expression differences in complex tissues.. Nat Methods.

[32] Koscielny G, Le Texier V, Gopalakrishnan C, Kumanduri V, Riethoven JJ (2009). ASTD: The Alternative Splicing and Transcript Diversity database.. Genomics.

[33] Feinberg AP, Tycko B (2004). The history of cancer epigenetics.. Nat Rev Cancer.

[34] Gordenin DA, Lobachev KS, Degtyareva NP, Malkova AL, Perkins E (1993). Inverted DNA repeats: a source of eukaryotic genomic instability.. Mol Cell Biol.

[35] Esteller M, Almouzni G (2005). How epigenetics integrates nuclear functions. Workshop on epigenetics and chromatin: transcriptional regulation and beyond.. EMBO Rep.

[36] Walsh CP, Chaillet JR, Bestor TH (1998). Transcription of IAP endogenous retroviruses is constrained by cytosine methylation.. Nat Genet.

[37] Dion V, Wilson JH (2009). Instability and chromatin structure of expanded trinucleotide repeats.. Trends Genet.

[38] Wang Y, Liska F, Gosele C, Sedova L, Kren V (2010). A novel active endogenous retrovirus family contributes to genome variability in rat inbred strains.. Genome Res.

[39] Lister R, Pelizzola M, Dowen RH, Hawkins RD, Hon G (2009). Human DNA methylomes at base resolution show widespread epigenomic differences.. Nature.

[40] Chodavarapu RK, Feng S, Bernatavichute YV, Chen PY, Stroud H (2010). Relationship between nucleosome positioning and DNA methylation.. Nature.

[41] Pandya-Jones A, Black DL (2009). Co-transcriptional splicing of constitutive and alternative exons.. RNA.

[42] Schwartz S, Meshorer E, Ast G (2009). Chromatin organization marks exon-intron structure.. Nat Struct Mol Biol.

[43] Schwartz S, Ast G (2010). Chromatin density and splicing destiny: on the cross-talk between chromatin structure and splicing.. EMBO J.

[44] Choi JK, Bae JB, Lyu J, Kim TY, Kim YJ (2009). Nucleosome deposition and DNA methylation at coding region boundaries.. Genome Biol.

[45] Anastasiadou C, Malousi A, Maglaveras N, Kouidou S (2011). Human epigenome data reveal increased CpG methylation in alternatively spliced sites and putative exonic splicing enhancers.. DNA Cell Biol.

[46] Luco RF, Allo M, Schor IE, Kornblihtt AR, Misteli T (2011). Epigenetics in alternative pre-mRNA splicing.. Cell.

[47] Brodsky AS, Meyer CA, Swinburne IA, Hall G, Keenan BJ (2005). Genomic mapping of RNA polymerase II reveals sites of co-transcriptional regulation in human cells.. Genome Biol.

[48] Yadav AK, Bhardwaj G, Basak T, Kumar D, Ahmad S (2011). A systematic analysis of eluted fraction of plasma post immunoaffinity depletion: implications in biomarker discovery.. PLoS One.

