# Supplementary figures and images for: High Resolution Methylome Map of Rat Indicates Role of Intragenic DNA Methylation in Identification of Coding Region
Source: PLoS One. 2012 Feb 15;7(2):e31621. doi: 10.1371/journal.pone.0031621 (PMC3280313; doi:10.1371/journal.pone.0031621)

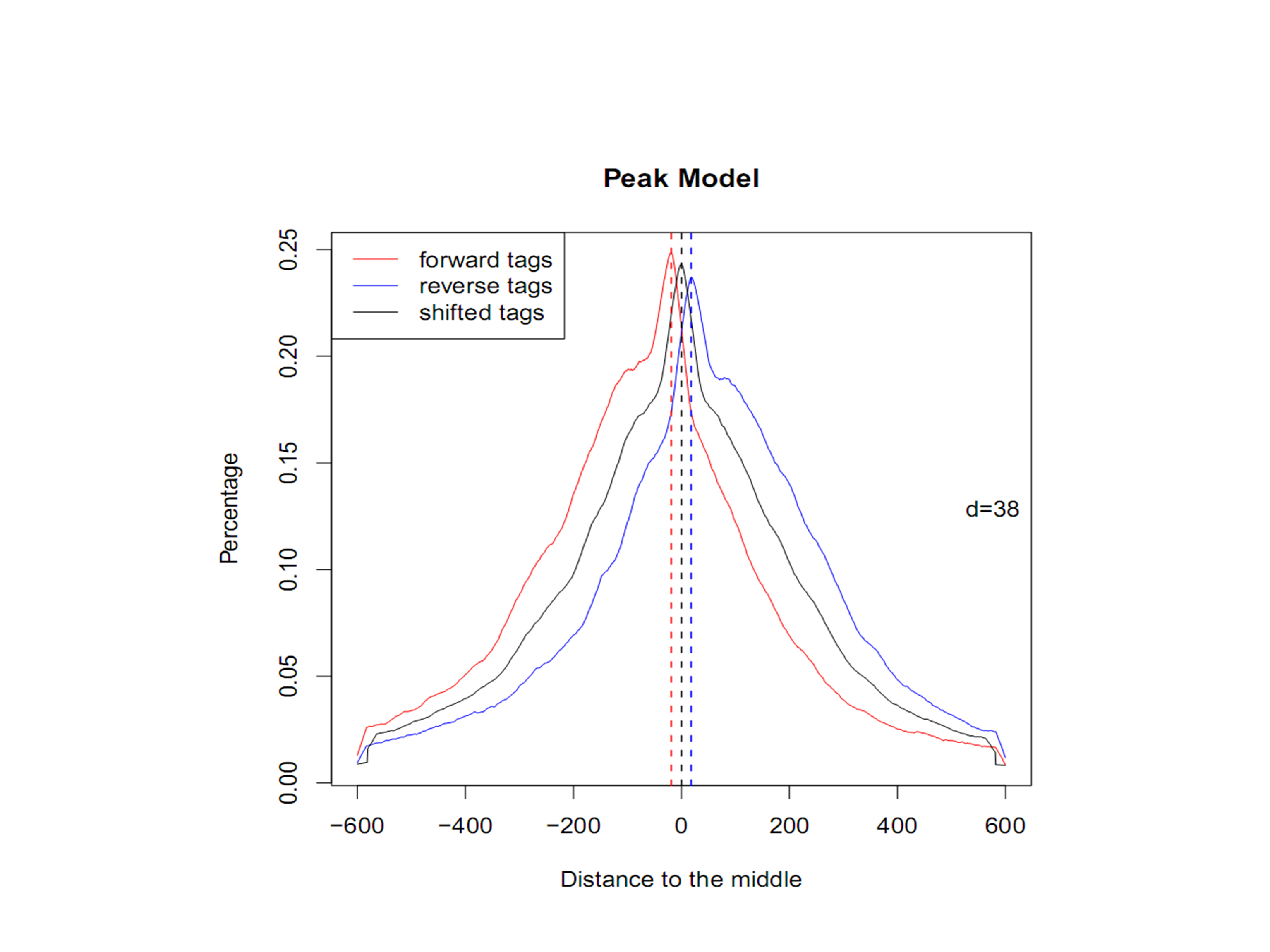

Supplement: Figure S1 — MACS model for MeDIP-Seq data. The reads generated from the MeDIP sequencing was processed through MACS (Model-based Analysis for ChIP-Seq) software version 1.4.0 beta for the generation of MACS model. The fragment size was 200 bp and the distance d between the forward and the reverse tags is 38. (TIF) [file pone.0031621.s001.tif]

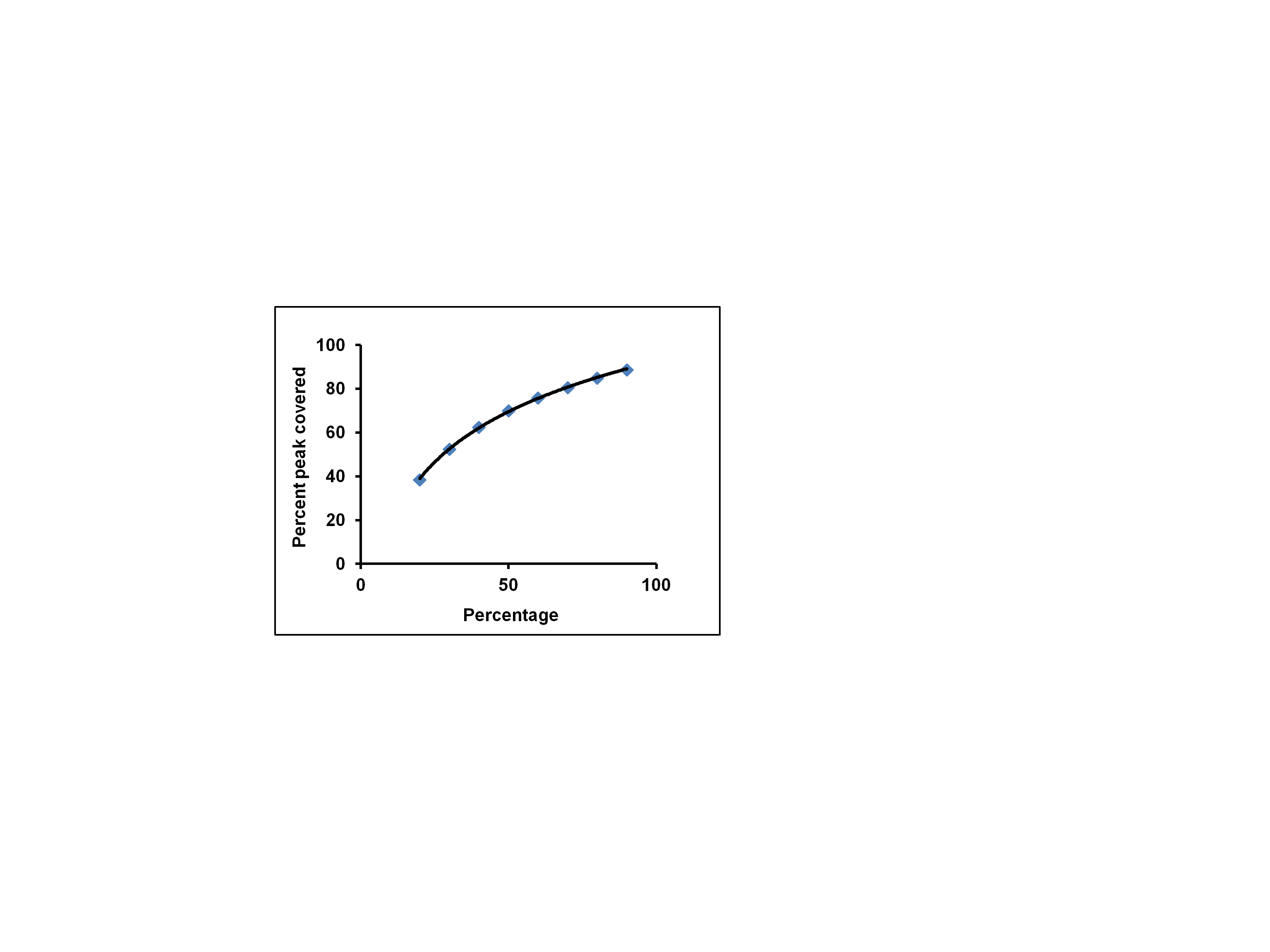

Supplement: Figure S2 — Saturation curve. The curve shows saturation at the end on plotting the percentage of peak covered by sampling (y-axis) against the percentage of raw reads take during data reduction approach (x-axis). (TIF) [file pone.0031621.s002.tif]

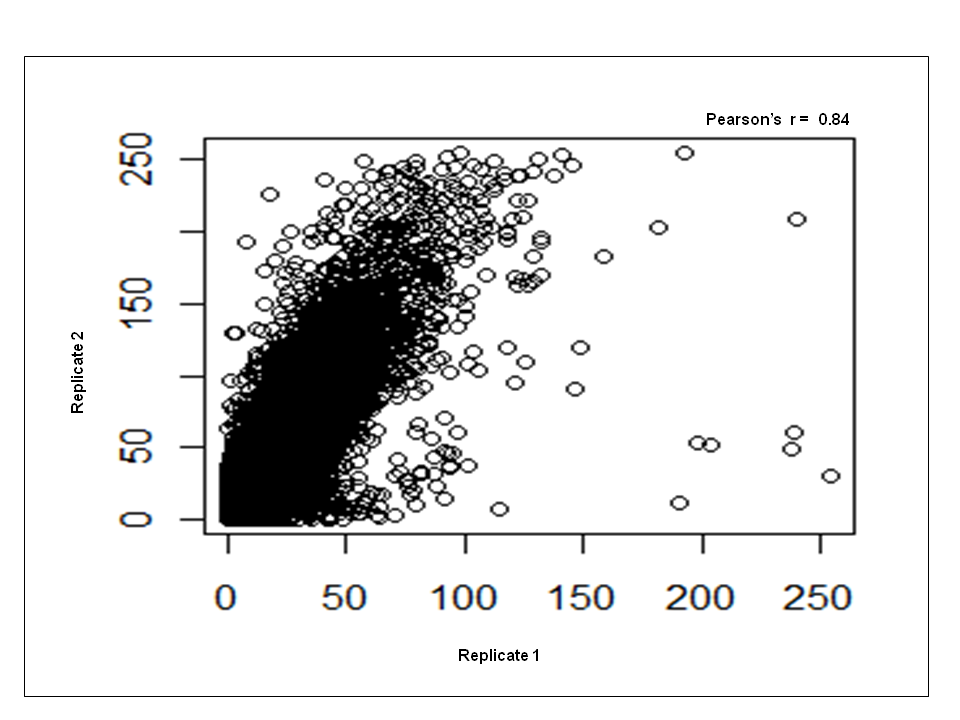

Supplement: Figure S3 — Concordance between replicates. Read coverage at each base pair was calculated separately for both the replicates and then Pearson's correlation was calculated using R programming and statistical language. (TIF) [file pone.0031621.s003.tif]

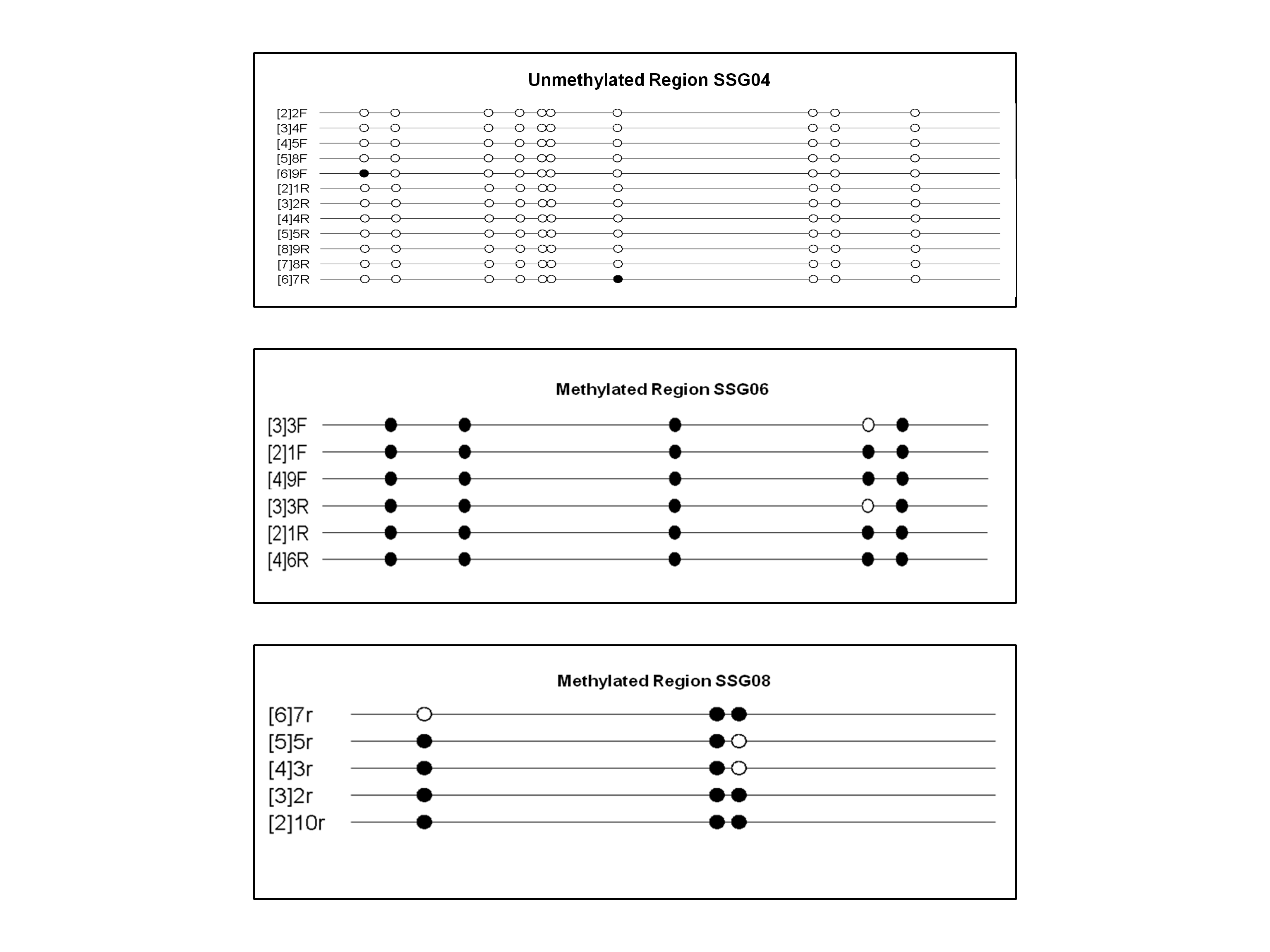

Supplement: Figure S4 — Bisulfite validation of MeDIP Seq data. Two regions (SSG06 and SSG08) showing high methylation and one region showing no methylation (SSG04) but with a number of CpGs were sequenced after bisulfite conversion and PCR amplification. (TIF) [file pone.0031621.s004.tif]

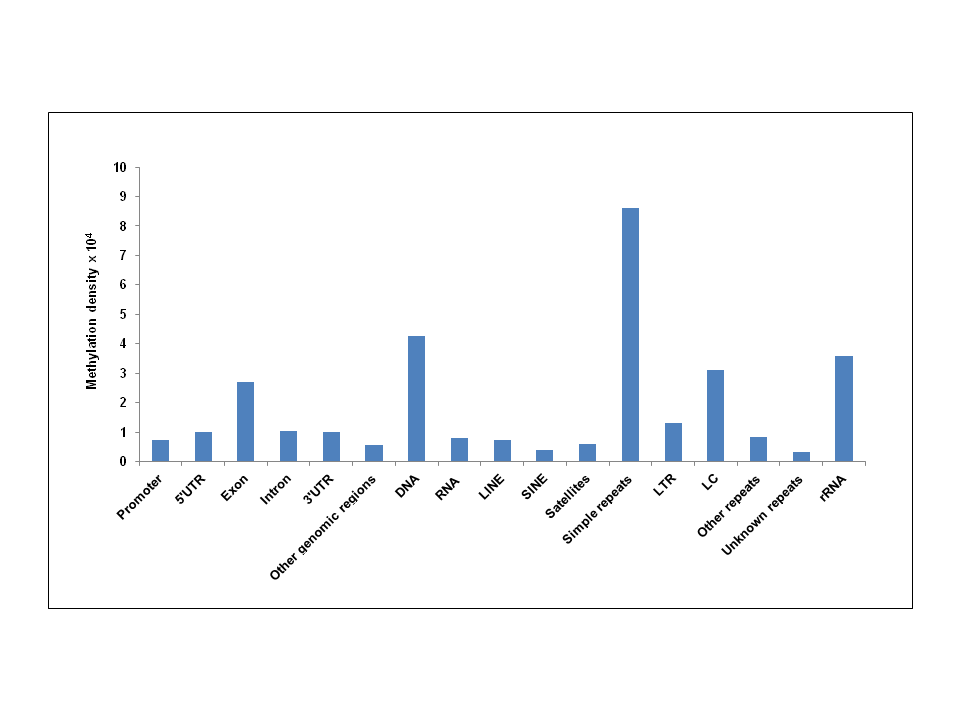

Supplement: Figure S5 — Methylation density in different bins. Methylation density within genomic features along with all the repeat class (DNA, RNA, LTR, LC, LINE, SINE, SATELLITE, SIMPLE REPEAT, OTHER REPEATS, and UNKNOWN REPEATS) and rRNA (a component of RNA class) was calculated. Genomic features include RefSeq exons, introns, identified liver expressed gene exons and introns. (TIF) [file pone.0031621.s005.tif]

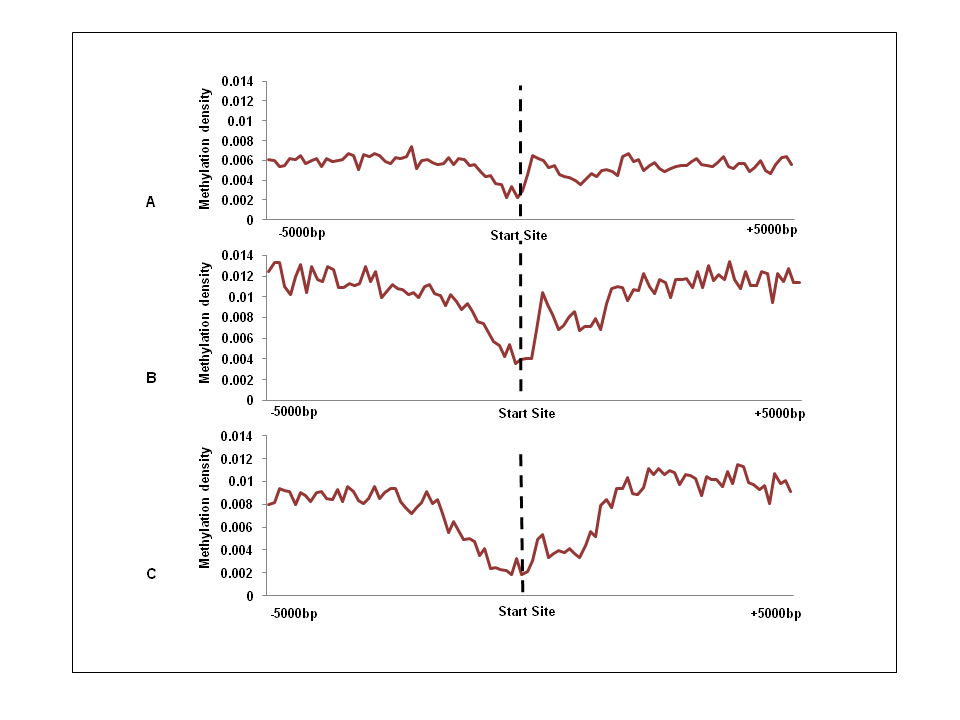

Supplement: Figure S6 — CpG methylation distribution around TSS in Human, mice and rats. Average methylation density around Transcription Start Site (TSS) of 3 different species. Distribution of peak summit count in 100 bp sliding window, 5 kb upstream and downstream from the start site was calculated for all RefSeq genes of A – Human and B - Mouse. Smoothing of peaks was done by taking moving average of 5. (TIF) [file pone.0031621.s006.tif]

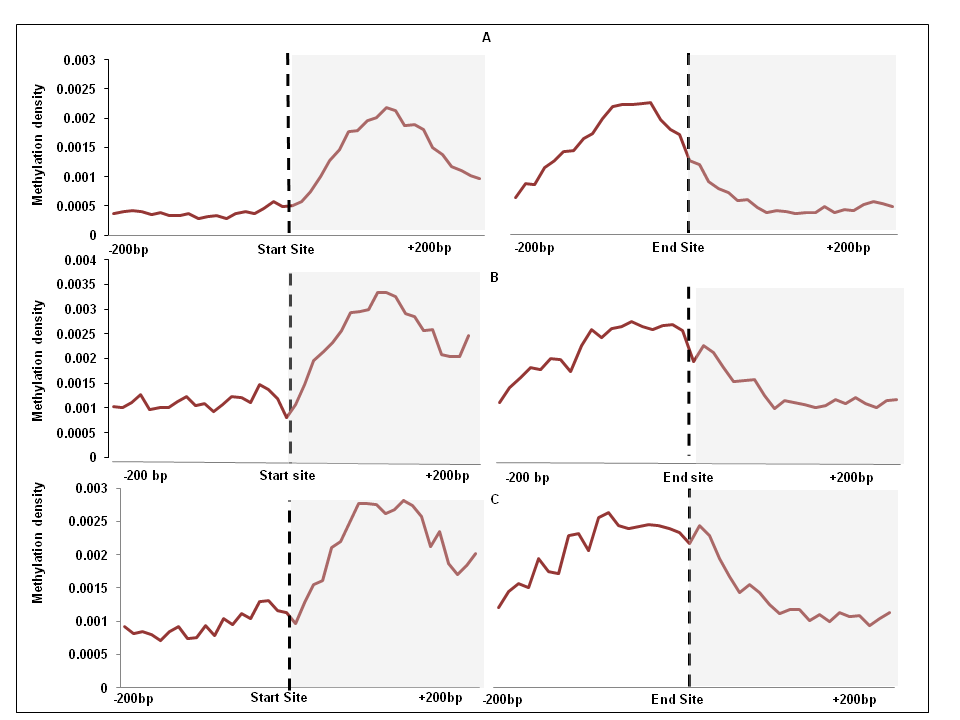

Supplement: Figure S7 — CpG methylation distribution at exon boundaries in Human, mice and rats. Methylation density around Exon/Intron junction of 3 different species. Distribution of peak summit count in 10 bp sliding window, 200 bp upstream and downstream from the start site was calculated for all exons of A- Human and B– Mouse. Smoothing of peaks was done by taking moving average of 5. (TIF) [file pone.0031621.s007.tif]

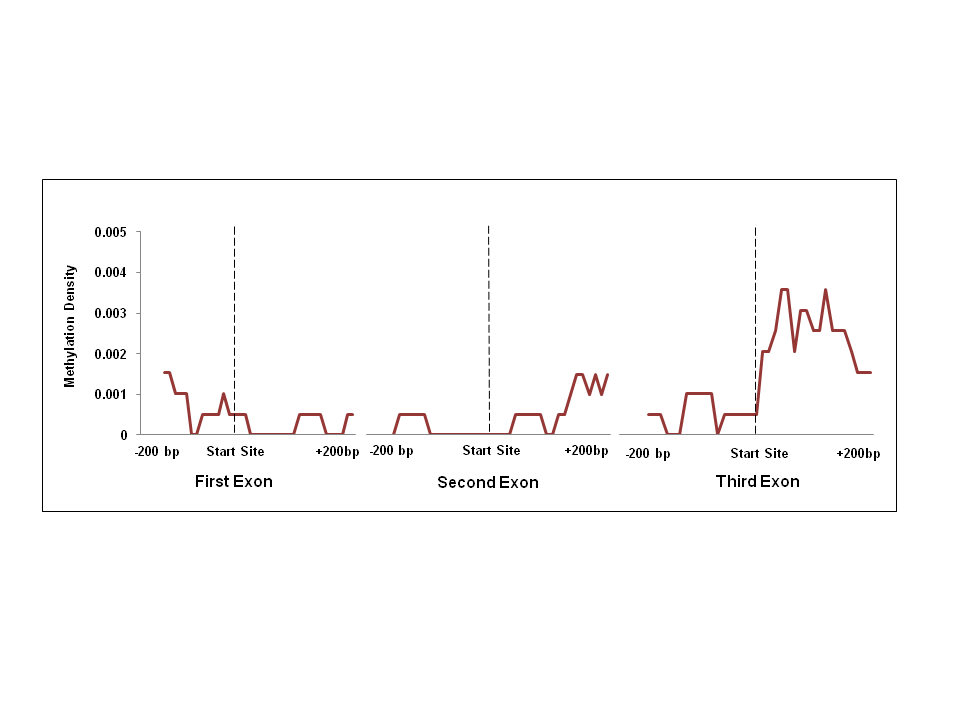

Supplement: Figure S8 — Methylation density at the start site of non coding 2nd exons. Distribution of peak summit count in 10 bp sliding window, 200 bp upstream and downstream from the start site was calculated for all 2nd exons which were non coding. Smoothing of peaks was done by taking moving average of 5. (TIF) [file pone.0031621.s008.tif]

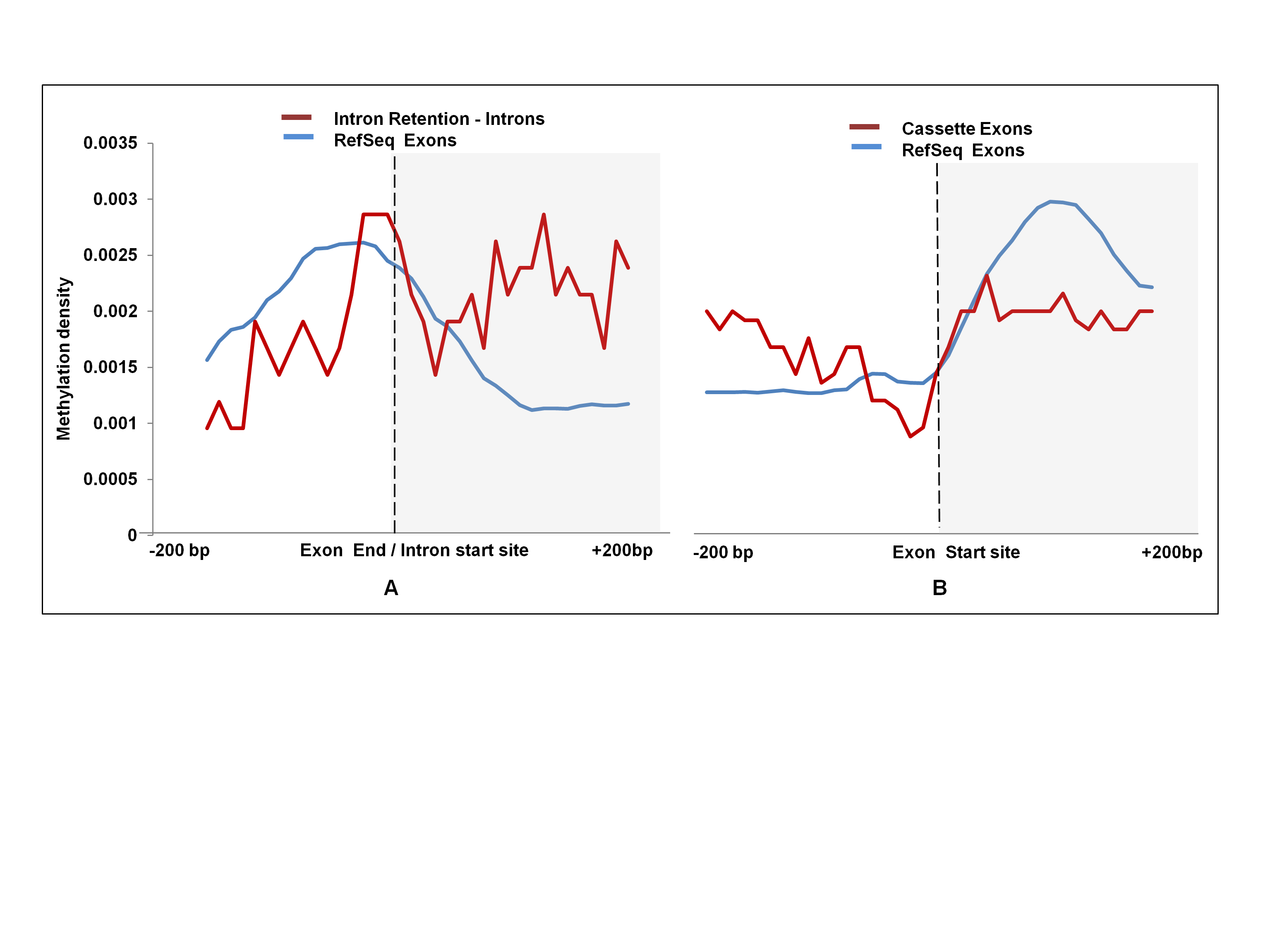

Supplement: Figure S9 — Methylation distribution in alternate splice events. CpG methylation distribution in two different alternate splice events. A: Distribution of peak summit count in 10 bp sliding window, 200 bp upstream and downstream from the start site of all RefSeq exons and cassette exons. Smoothing of peaks was done by taking moving average of 5. B: Distribution of peak summit count in 10 bp sliding window, 200 bp upstream and downstream from the end site of RefSeq exons and introns of Intron Retention class. Smoothing of peaks was done by taking moving average of 5. (TIF) [file pone.0031621.s009.tif]

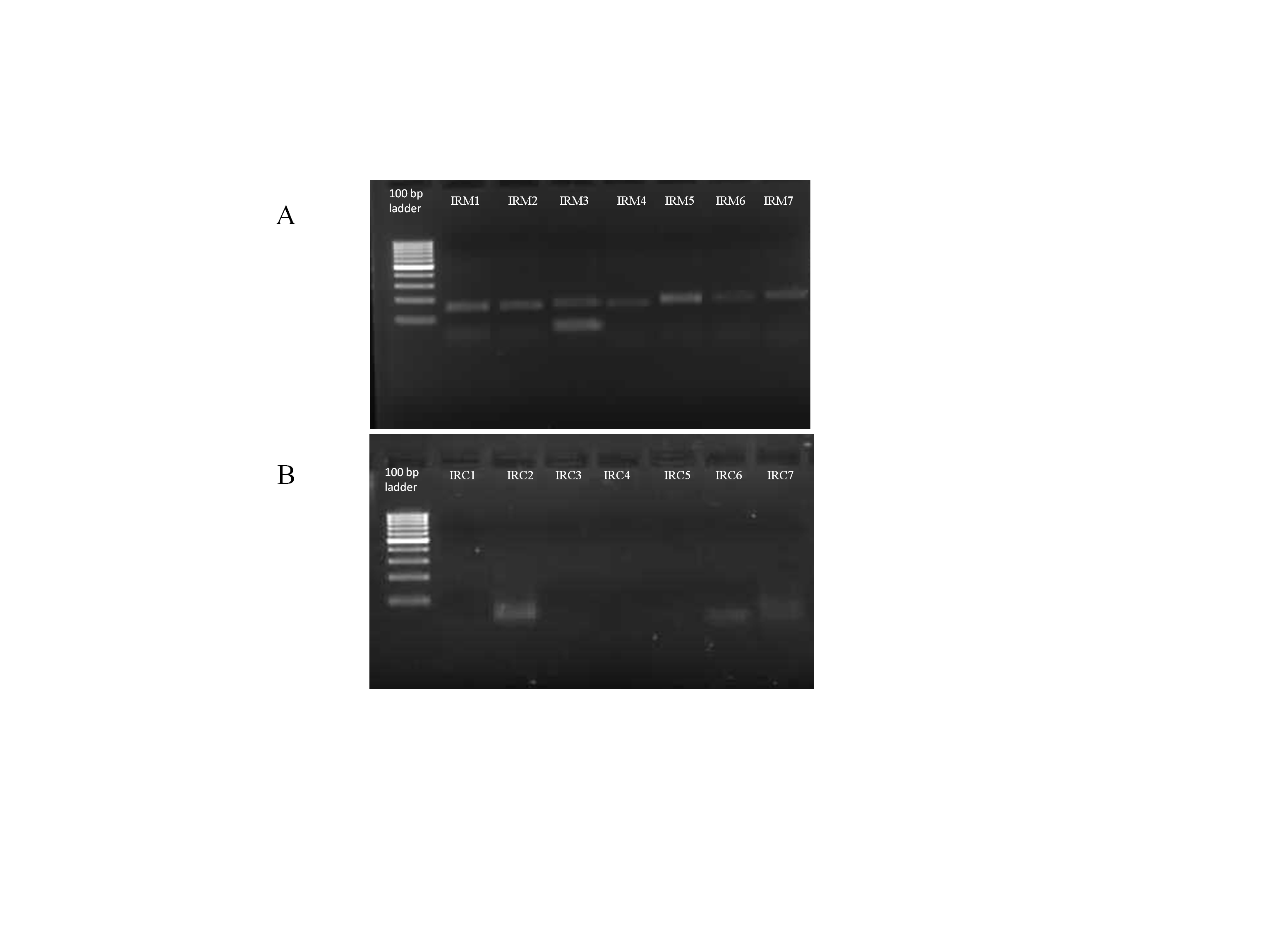

Supplement: Figure S10 — PCR amplification products of alternate splice events. PCR amplification of introns of IR category showing methylation in our MeDIP-Seq data (A; IRM1 to IRM7) and their constitutive counterparts (B; IRC1 to IRC7). (TIF) [file pone.0031621.s010.tif]

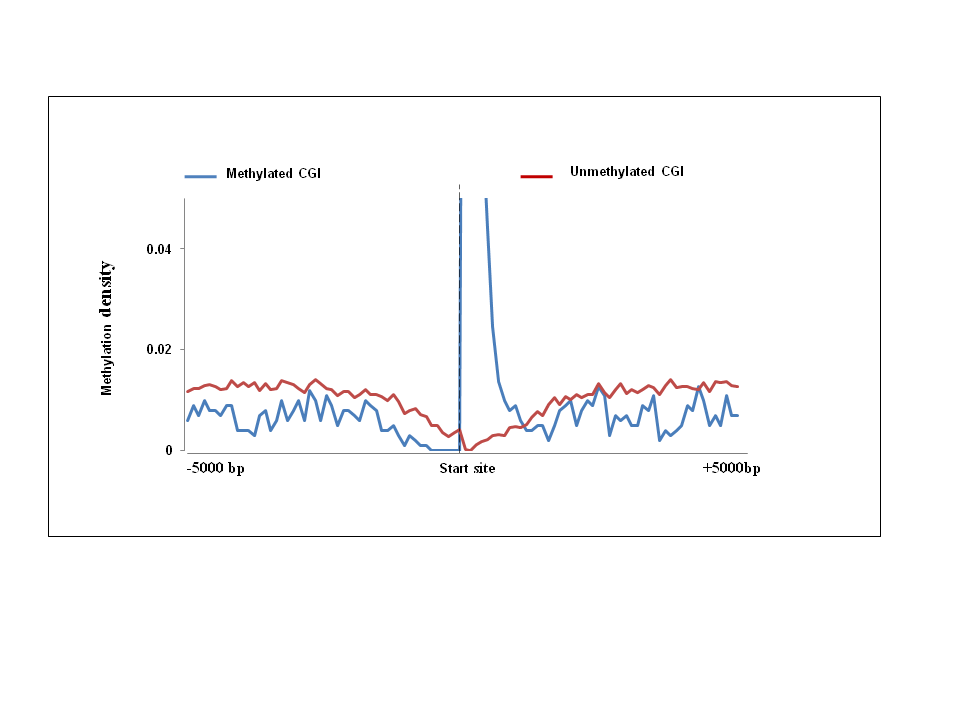

Supplement: Figure S11 — Methylated and Unmethylated CGI distribution around TSS. Methylation pattern in methylated/unmethylated CpG Island around their start site. Distribution of peak summit count in 100 bp sliding window, 5 kb upstream and downstream from the start site was calculated for methylated and unmethylated CpG Islands. (TIF) [file pone.0031621.s011.tif]

**File S1: Chromosomal distribution of methylation**

**
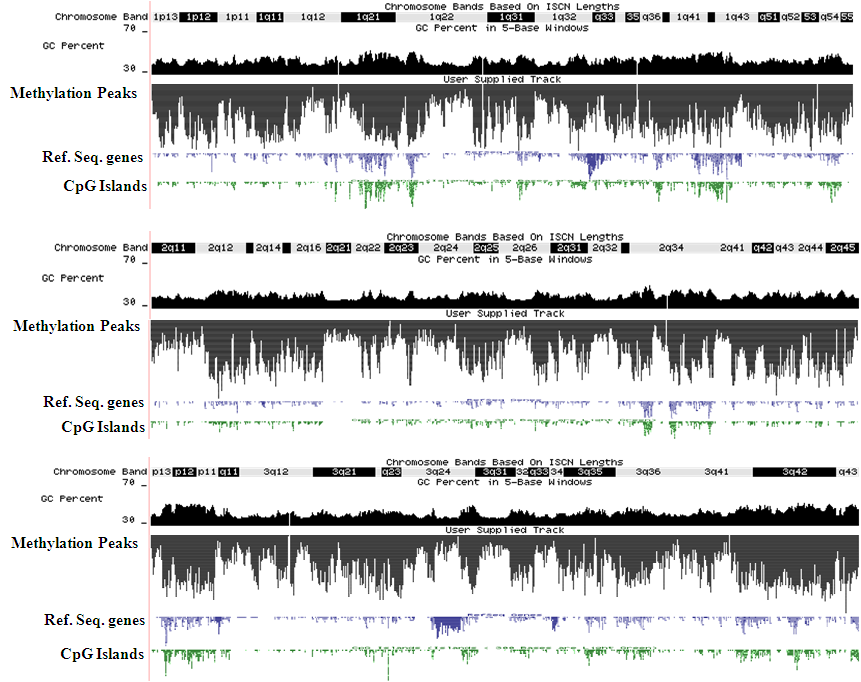
**

**
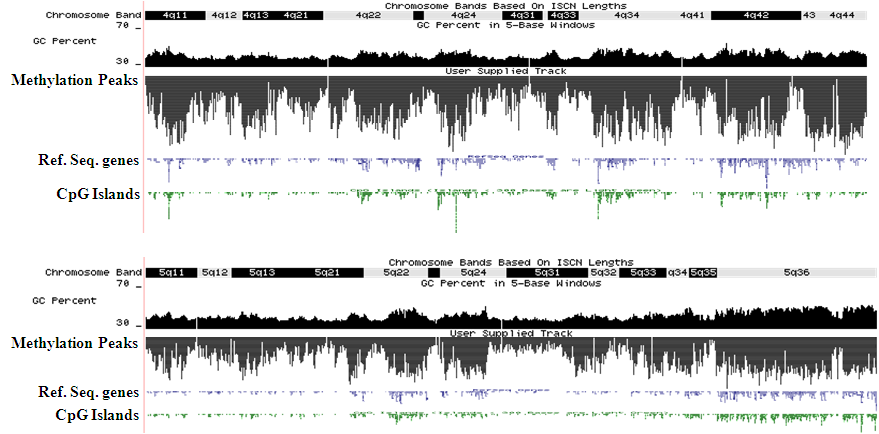
**

**
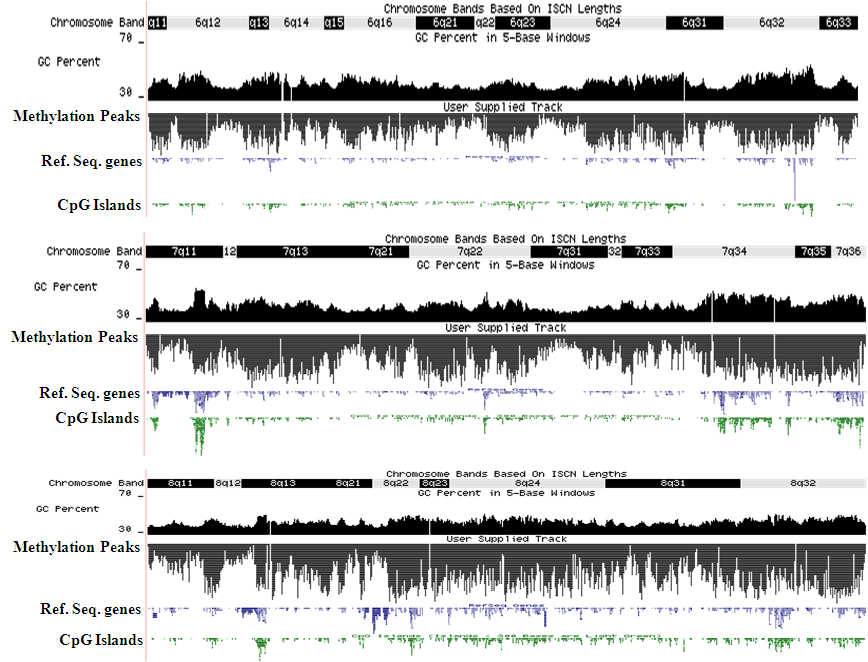
**

**
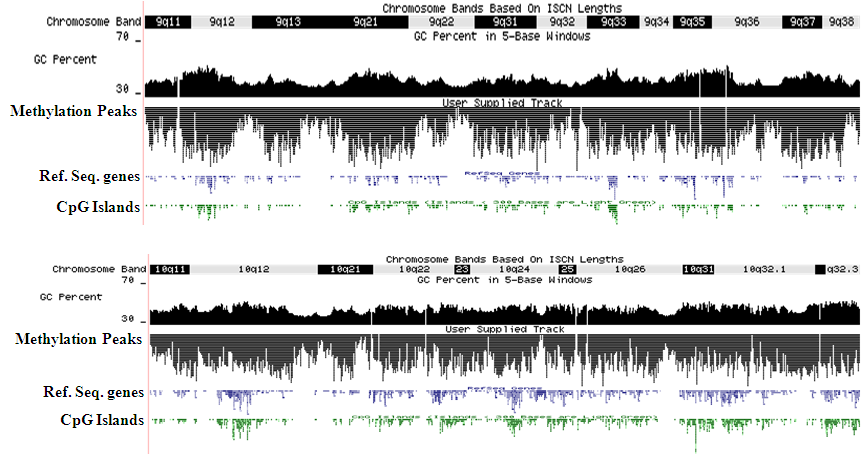
**

**
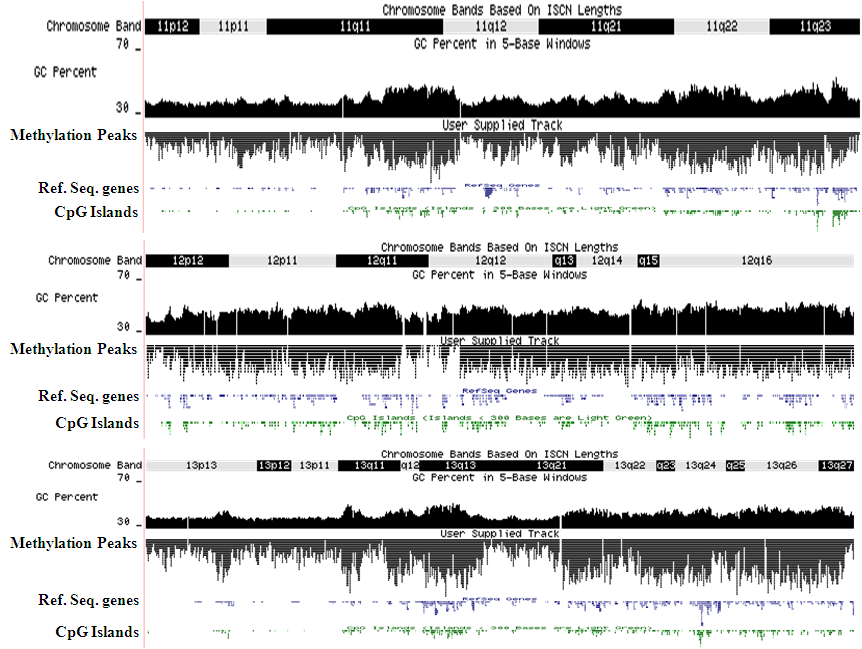
**

**
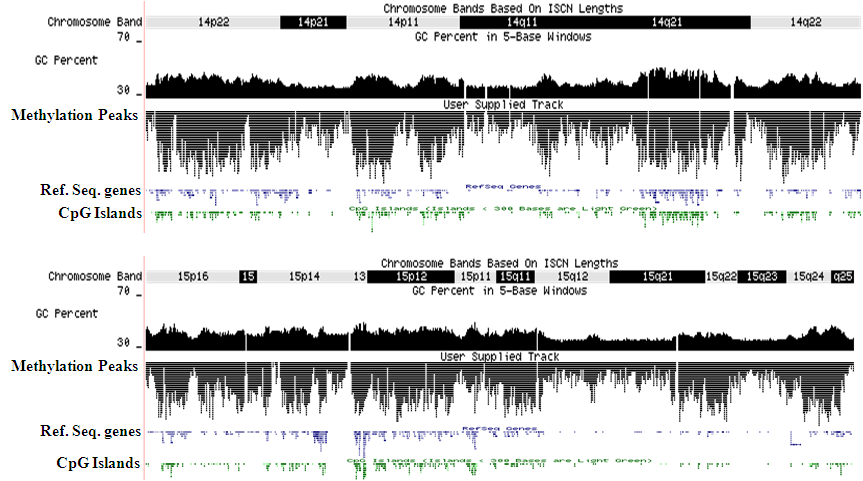
**

**
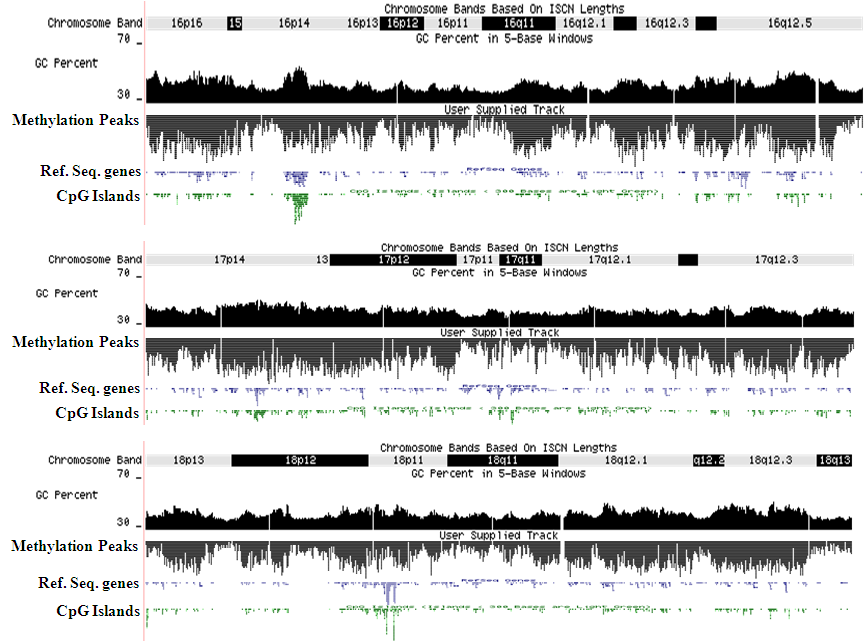
**

**
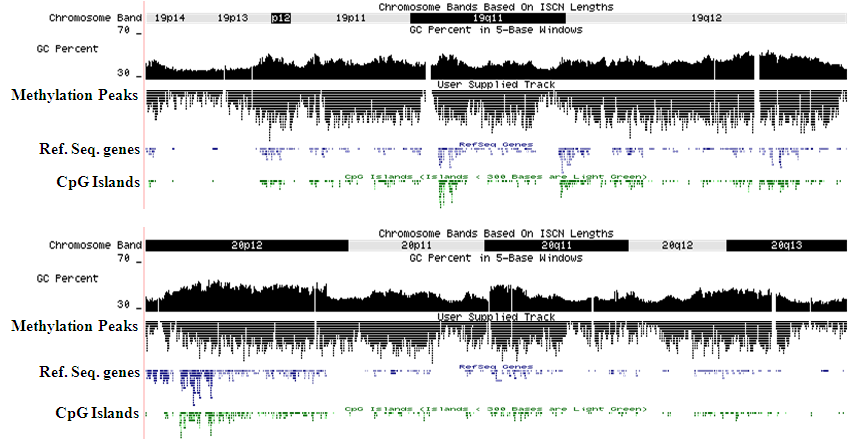
**

**
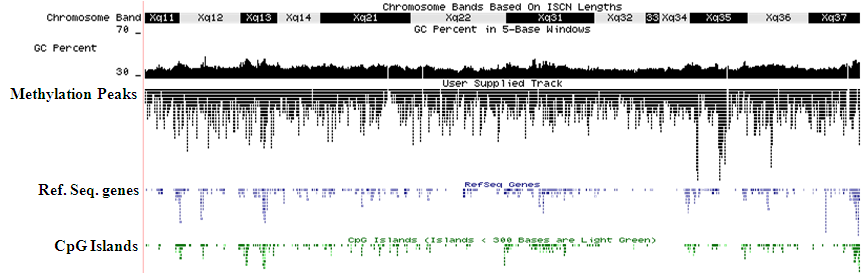
**

Supplement: File S1 — Chromosomal distribution of methylation. The methylation tracks visualized in UCSC genome browser with CGI tracks and RefSeq genes for all chromosomes. (DOCX) [file pone.0031621.s016.docx]
