# Supplementary material for: High Resolution Methylome Map of Rat Indicates Role of Intragenic DNA Methylation in Identification of Coding Region
Source: PLoS One. 2012 Feb 15;7(2):e31621. doi: 10.1371/journal.pone.0031621 (PMC3280313; doi:10.1371/journal.pone.0031621)
Supplement: Table S1 — Real Time PCR validation of the MeDIP process. The methylated regions were selected as imprinted regions either in rat (H19) or in Human and mice (Gnas). Negative regions were randomly taken from the genome where there were no CpGs. The enrichment is shown as E. (DOCX) [file pone.0031621.s012.docx]

**Table S1**: **Real Time PCR validation of the MeDIP process.**

| **Primer Code** | **Average Cp Value (MeDIP fraction)** | **Average Cp Value (Input fraction)** | **∆Ct** | **E = 2^ΔΔCt^** |
| --- | --- | --- | --- | --- |
| **Gnas 2 (Methylated)** | 22.66 | 28.58 | 5.92 | 7.49 (Chr13) and 8.91 (Chr14) |
| **H19 3.3 (Methylated)** | 23.71 | 27.07 | 3.36 | 4.93 (Chr13) and 6.35 (chr14) |
| **Chr 13 (Unmethylated)** | 28.75 | 27.18 | -1.57 |  |
| **Chr 14 (Unmethylated)** | 31.05 | 28.36 | -2.99 |  |
