# Supplementary material for: High Resolution Methylome Map of Rat Indicates Role of Intragenic DNA Methylation in Identification of Coding Region
Source: PLoS One. 2012 Feb 15;7(2):e31621. doi: 10.1371/journal.pone.0031621 (PMC3280313; doi:10.1371/journal.pone.0031621)
Supplement: Table S2 — Table showing percentage of reads versus percentage of peaks called by MACS. Table showing the data generated by MACS employing data reduction approach after the model generation. (DOCX) [file pone.0031621.s013.docx]

**Table S2**: **Table showing percentage of reads versus percentage of peaks called by MACS**

| **Percentage** | **Percentage of peak covered** |
| --- | --- |
| 20 | 38.61 |
| 30 | 52.55 |
| 40 | 62.62 |
| 50 | 70.1 |
| 60 | 76.01 |
| 70 | 80.74 |
| 80 | 84.98 |
| 90 | 88.76 |
