# Supplementary material for: High Resolution Methylome Map of Rat Indicates Role of Intragenic DNA Methylation in Identification of Coding Region
Source: PLoS One. 2012 Feb 15;7(2):e31621. doi: 10.1371/journal.pone.0031621 (PMC3280313; doi:10.1371/journal.pone.0031621)
Supplement: Table S3 — Distribution of CGI based on size. Distribution of mCGI and uCGI based on their size and genomic location. (DOCX) [file pone.0031621.s014.docx]

**Table S3: Distribution of CpG Islands based on size.**

| **Type** | **Promoter** | | **Exon** | | **Intron** | | **UTR** | | **Other** | | **Total CGI** | |
| --- | --- | --- | --- | --- | --- | --- | --- | --- | --- | --- | --- | --- |
| **200-300** | 30 | 790 | 156 | 582 | 95 | 699 | 16 | 365 | 189 | 578 | 486 | 3014 |
| **300-400** | 16 | 763 | 68 | 381 | 36 | 574 | 10 | 321 | 57 | 352 | 187 | 2391 |
| **400-500** | 6 | 710 | 45 | 276 | 17 | 526 | 9 | 289 | 18 | 187 | 95 | 1988 |
| **500-600** | 3 | 606 | 24 | 185 | 6 | 497 | 1 | 211 | 28 | 131 | 62 | 1630 |
| **Above 600** | 28 | 2173 | 85 | 360 | 36 | 1814 | 19 | 831 | 22 | 593 | 190 | 5771 |

**Methylated CGI Unmethylated CGI**
