# Supplementary material for: High Resolution Methylome Map of Rat Indicates Role of Intragenic DNA Methylation in Identification of Coding Region
Source: PLoS One. 2012 Feb 15;7(2):e31621. doi: 10.1371/journal.pone.0031621 (PMC3280313; doi:10.1371/journal.pone.0031621)
Supplement: Table S4 — Primer list. List of primers used in the study: The list contains primers used for calculating MeDIP-Seq enrichment efficiency, for bisulfite PCR and those used for the reverse transcriptase PCR of the rat liver cDNA. (DOCX) [file pone.0031621.s015.docx]

**Table S4: Primer sequences**

| **S.No.** | **Primer Name** | **Process** | **Sequence** | **Annealing temperature** |
| --- | --- | --- | --- | --- |
| 1 | Gnas 1 F | MeDIP, Real time PCR | GTGAGCGAGCGTATCACATGC | 63.3 |
| 2 | Gnas 1 R | MeDIP, Real time PCR | CAGTGTTGATGCAAACAGAGCAAG | 63.5 |
| 3 | Plagl 1 F | MeDIP, Real time PCR | CTACCTATTCTCCTCCCTCATTG | 62.9 |
| 4 | Plagl 1 R | MeDIP, Real time PCR | GTGAGCGAGCGTATCACATGC | 62.9 |
| 5 | Chromosome 14 F | MeDIP, Real time PCR | ACAAAATCACTTAGTAAGGCACAGG | 62.5 |
| 6 | Chromosome 14 R | MeDIP, Real time PCR | GGACTAAAGCTACAGATGATTGGAA | 62.5 |
| 7 | Chromosome 13 F | MeDIP, Real time PCR | AAATAACCTCTAAGCAGGAATCTGGT | 62.5 |
| 8 | Chromosome 13 R | MeDIP, Real time PCR | CTGTAAACACCTTTGTAAGGGCTAA | 63.1 |
| 9 | SS004 F | Bisulfite PCR | ATAGGTTTTAAGTAGTGATGGATATAGT | 61.3 |
| 10 | SS004 R | Bisulfite PCR | TACTTCCCTCAACACTAAAAATTTAC | 60 |
| 11 | SS006 F | Bisulfite PCR | GGAGAATTGAATAGGGATAAAAGGT | 60.9 |
| 12 | SS006 R | Bisulfite PCR | TCTACCCACATTATAAAAACCCAAA | 59.2 |
| 13 | SS008 F | Bisulfite PCR | TGAAGAAGGGTTTAGTTTGGTGT | 59.3 |
| 14 | SS008 R | Bisulfite PCR | TAAACAACAAAAACCACTCACACAT | 59.2 |
| 15 | IRM 1 F | Intron retention | CAGGCTCCCAAGAATGAGTC | 59.8 |
| 16 | IRM 1 R | Intron retention | GGTCCAGCTCTGCCTACATC | 59.8 |
| 17 | IRM 2 F | Intron retention | CAGGCTCCCAAGAATGAGTC | 59.8 |
| 18 | IRM 2 R | Intron retention | GGTCCAGCTCTGCCTACATC | 59.8 |
| 19 | IRM 3 F | Intron retention | GCAGGTCAGGAGTTCTTCCA | 60.4 |
| 20 | IRM 3 R | Intron retention | CGTCCCCAAGAAGAACAGAT | 59.1 |
| 21 | IRM 4 F | Intron retention | TCCTTCCAGTCTCCAGATCAA | 59.7 |
| 22 | IRM 4 R | Intron retention | CAGCAGGAGGGAAGACTGAC | 60 |
| 23 | IRM 5 F | Intron retention | AGCAGATTCCTGGGAGATGA | 59.7 |
| 24 | IRM 5 R | Intron retention | GGCGTAACATGTTGGACACA | 60.4 |
| 25 | IRM 6 F | Intron retention | AAGCCGCACAGAGGAAATTA | 59.8 |
| 26 | IRM 7 R | Intron retention | AGGCACCGTGGTAACTTGAG | 60.1 |
| 27 | IRM 7 F | Intron retention | AAGGAGTGATGTGCCAGGAG | 60.2 |
| 28 | IRM 7 R | Intron retention | GGGGTAGTCCTGCAAAGACA | 60.1 |
| 29 | IRC 1 F | Intron retention | CGGTTAGGTTGAGTGGGAAG | 60.5 |
| 30 | IRC 1 R | Intron retention | GCCACGACTACTGGGAAGAG | 62..5 |
| 31 | IRC 2 F | Intron retention | GGGTGGGTGAGTGAGTGAAT | 60.5 |
| 32 | IRC 2 R | Intron retention | ACGCCTGCAGAGAACAAAGT | 58.4 |
| 33 | IRC 3 F | Intron retention | TGAAAGTGCTGCAGGAAGTG | 58.4 |
| 34 | IRC 3 R | Intron retention | CGCTGAGCCAGTTTAAGCA | 57.5 |
| 35 | IRC 4 F | Intron retention | CCTGGAAGGAGGGGACTTAG | 62.5 |
| 36 | IRC 4 R | Intron retention | AGGCAGGGACATTCAGTCAC | 60.5 |
| 37 | IRC 5 F | Intron retention | ATCTGGCTACACGGGCTATG | 60.5 |
| 38 | IRC 5 R | Intron retention | CCGCCTCCTACTACAACCTG | 62.5 |
| 39 | IRC 6 F | Intron retention | ACCTACTCAGAAGCGTGCAG | 60.5 |
| 40 | IRC 6 R | Intron retention | TGGCAACGGTTGAAATTGTA | 59.5 |
| 41 | IRC 7 F | Intron retention | TCCGTTCTGGAGACTGGAGTA | 61.3 |
| 42 | IRC 7 R | Intron retention | AATGTACCATCCAGCACAGC | 58.4 |
